# Not All Lesioned Tissue Is Equal: Identifying Pericavitational Areas in Chronic Stroke With Tissue Integrity Gradation *via* T2w T1w Ratio

**DOI:** 10.3389/fnins.2021.665707

**Published:** 2021-08-05

**Authors:** Lisa C. Krishnamurthy, Venkatagiri Krishnamurthy, Amy D. Rodriguez, Keith M. McGregor, Clara N. Glassman, Gabriell S. Champion, Natalie Rocha, Stacy M. Harnish, Samir R. Belagaje, Suprateek Kundu, Bruce A. Crosson

**Affiliations:** ^1^Center for Visual and Neurocognitive Rehabilitation, Atlanta VA Health Care System, Decatur, GA, United States; ^2^Department of Physics and Astronomy, Georgia State University, Atlanta, GA, United States; ^3^Division of Geriatrics and Gerontology, Department of Medicine, Emory University, Atlanta, GA, United States; ^4^Department of Neurology, Emory University, Atlanta, GA, United States; ^5^Department of Nuclear and Radiological Engineering and Medical Physics, Georgia Institute of Technology, Atlanta, GA, United States; ^6^Department of Psychology, Georgia State University, Atlanta, GA, United States; ^7^Department of Speech and Hearing Science, The Ohio State University, Columbus, OH, United States; ^8^Department of Rehabilitation Medicine, Emory University, Atlanta, GA, United States; ^9^Department of Biostatistics and Bioinformatics, Emory University, Atlanta, GA, United States

**Keywords:** stroke lesion, TIGR, pericavitational area, task fMRI, cerebral blood flow

## Abstract

Stroke-related tissue damage within lesioned brain areas is topologically non-uniform and has underlying tissue composition changes that may have important implications for rehabilitation. However, we know of no uniformly accepted, objective non-invasive methodology to identify pericavitational areas within the chronic stroke lesion. To fill this gap, we propose a novel magnetic resonance imaging (MRI) methodology to objectively quantify the lesion core and surrounding pericavitational perimeter, which we call tissue integrity gradation *via* T2w T1w ratio (TIGR). TIGR uses standard T1-weighted (T1w) and T2-weighted (T2w) anatomical images routinely collected in the clinical setting. TIGR maps are analyzed with relation to subject-specific gray matter and cerebrospinal fluid thresholds and binned to create a false colormap of tissue damage within the stroke lesion, and these are further categorized into low-, medium-, and high-damage areas. We validate TIGR by showing that the cerebral blood flow within the lesion reduces with greater tissue damage (*p* = 0.005). We further show that a significant task activity can be detected in pericavitational areas and that medium-damage areas contain a significantly lower magnitude of hemodynamic response function than the adjacent damaged areas (*p* < 0.0001). We also demonstrate the feasibility of using TIGR maps to extract multivariate brain–behavior relationships (*p* < 0.05) and show general agreement in location compared to binary lesion, T1w-only, and T2w-only maps but that the extent of brain behavior maps may depend on signal sensitivity as denoted by the sparseness coefficient (*p* < 0.0001). Finally, we show the feasibility of quantifying TIGR in early and late subacute stroke phases, where higher-damage areas were smaller in size (*p* = 0.002) and that lesioned voxels transition from lower to higher damage with increasing time post-stroke (*p* = 0.004). We conclude that TIGR is able to (1) identify tissue damage gradient within the stroke lesion across different post-stroke timepoints and (2) more objectively delineate lesion core from pericavitational areas wherein such areas demonstrate reasonable and expected physiological and functional impairments. Importantly, because T1w and T2w scans are routinely collected in the clinic, TIGR maps can be readily incorporated in clinical settings without additional imaging costs or patient burden to facilitate decision processes related to rehabilitation planning.

## Introduction

Histological evidence points to a gradient of damage within stroke lesions (Liebeskind, 2003; Bang et al., 2008; Sofroniew, 2009; Campbell et al., 2013; Anderson et al., 2014; Burda and Sofroniew, 2014; Adams and Gallo, 2018), leading to underlying tissue composition changes that shape cortical reorganization (Brown et al., 2008). A challenge to rehabilitation after stroke is non-invasively identifying the “pericavitational” perimeter, defined as damaged tissue within the lesion that may still contain living cell bodies (Anderson et al., 2014; Burda and Sofroniew, 2014; Adams and Gallo, 2018). The pericavitational perimeter is an important potential resource for rehabilitation because functional recovery can stimulate re-sprouting of neural dendrites that were lost due to stroke (Brown et al., 2007). Remapping of pericavitational areas may depend on reengaging damaged yet viable areas (Murphy and Corbett, 2009), especially because lost function is more likely remapped to pericavitational areas that encode similar functions (Brown et al., 2009). Therefore, we envision that non-invasive identification of viable pericavitational areas may provide a new set of targets to engage during stroke rehabilitation.

Multiple factors can promote stroke recovery, including treatments that foster neuroplasticity (Laska et al., 2001; Hamilton et al., 2011), non-invasive brain stimulation and behavioral manipulations that engage or suppress specific cortices (Hamilton et al., 2011; Crosson et al., 2019), and recruitment of lesioned and perilesional brain regions (Hamilton et al., 2011). Some studies suggest that perilesional areas reacquire function in the weeks and months following an injury. In animal models of acute stroke, neuroplasticity was enhanced in perilesional areas where neural sprouting occurred (Brown et al., 2007, 2009; Murphy and Corbett, 2009).

To harness the rehabilitation potential of the pericavitational perimeter, these areas must first be identified. We know of no universally accepted objective non-invasive method to identify pericavitational areas within a lesion, representing a significant gap to understanding and possibly improving stroke recovery. T1-weighted (T1w) and T2-weighted (T2w) anatomical magnetic resonance imaging (MRI) scans show signal changes in chronic stroke lesions compared to healthy tissue. Stroke-induced changes of brain tissue composition have a direct and pronounced impact on tissue T1 and T2, either because the water content has increased (fluid-filled cyst) or because the underlying cellular profile has changed (demyelination, Wallerian degeneration, and proliferation of reactive astrocytes and microglia). In effect, high-resolution T1w and T2w anatomical MRI scans are good candidates to non-invasively detect and classify gradients of tissue damage within the chronic stroke lesion and have the potential to non-invasively classify cavitation *versus* pericavitational perimeter.

Most stroke studies use a binary approach to delineate damaged areas as compared to spared regions or, stated simply: brain regions are treated as essentially good or gone. In this study, we develop a novel method, called tissue integrity gradation *via* T2w T1w ratio (TIGR), to identify pericavitational areas in chronic stroke lesions. We hypothesize that TIGR can identify pericavitational areas in chronic stroke regions and that cerebral blood flow (CBF) correlates with TIGR-classified tissue damage, thereby non-invasively validating TIGR. We also hypothesize that a significant task functional MRI (fMRI) activity can be observed in pericavitational areas identified by TIGR. We also hypothesize that TIGR can be used to generate brain–behavior maps. Finally, although developed in chronic stroke lesions, we hypothesize that TIGR can be applied to early and late subacute stroke lesions.

## Materials and Methods

### General Procedures

The data were from 35 mono-lingual English-speaking subjects with aphasia (age range, 35–92 years old) who were >6 months post left-hemisphere stroke (range of time since stroke, 0.8–17.3 years). The participants were excluded if they presented with a history of mental health disorders or other neurological disorders. Evidence of aphasia was determined by Western Aphasia Battery—Revised (WAB) AQ (range, 27.4–96.8) and clinical impressions of the strengths and weaknesses in language function of the subjects as judged by experienced and certified speech–language pathologists (ADR or SMH; see Supplementary Table 1). The subjects participated in an MRI session and a language assessment session which included the administration of the WAB (Kertesz, 2007). The raw scores from the WAB Spontaneous Speech, Auditory Comprehension, and Repetition subtests were used as the continuous language behavior scores in this study.

### High-Resolution Anatomical MRI Acquisition

T1-weighted and T2-weighted MRI scans were acquired on either a 3-T Philips Achieva MRI scanner (Best, Netherlands) using the body coil for radio frequency (RF) transmission and an eight-channel phased-array head coil for RF receiving or a 3-T Siemens Prisma MRI scanner (Erlangen, Germany) using the body coil for RF transmission and a 32-channel phased-array head coil for RF receiving. It is feasible to combine data from two MRI scanners because of the thresholding and normalization procedures incorporated in the TIGR methodology as described below.

Two types of high-resolution anatomical MRI scans were acquired on each subject: (1) a T1-weighted high-resolution anatomical image and (2) a T2-weighted high-resolution anatomical image. The T1w high-resolution anatomical image incorporated the following sequence parameters on the Siemens Prisma: T1-MPRAGE, TR = 2,530 ms, TE = 2.96 ms, TI = 1,100 ms, FA = 7°, isotropic resolution = 1 mm × 1 mm × 1 mm, acquisition bandwidth = 130 Hz; or on the Philips Achieva: T1-TFE, TR = 2,390 ms, TE = 3.69 ms, TI = 1,100 ms, FA = 8°, resolution = 0.94 mm × 0.94 mm × 1 mm, acquisition bandwidth = 191 Hz. The T2w high-resolution anatomical image had the following acquisition parameters on the Siemens Prisma: T2-SPACE, TR = 3,200 ms, TE = 285 ms, FA = 120°, isotropic resolution = 1 mm × 1 mm × 1 mm, acquisition bandwidth = 700 Hz; or on the Philips Achieva: TSE, TR = 2,500 ms, TE = 399 ms, FA = 90°, resolution = 1 mm × 1 mm × 1 mm, acquisition bandwidth = 868 Hz.

### Calculation of TIGR Maps

**FIGURE 1:**
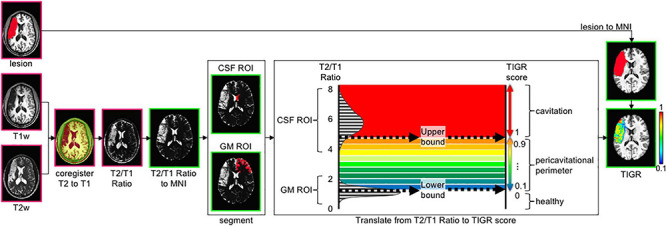
The tissue integrity gradation *via* T2w T1w ratio (TIGR) analysis pipeline. The user input includes a T1w image, T2w image, and a binary lesion mask. After co-registering the T2w and T1w images, the T2w/T1w ratio is computed on a voxel-wise basis and transformed into MNI template space. The right hemisphere anterior lateral ventricle is segmented to represent the cerebrospinal fluid (CSF) region of interest (ROI), and the anterior contralateral gray matter (GM) ribbon is segmented to represent the GM ROI. The histogram of T2w/T1w signal distribution is plotted for each individual subject and then thresholded as described in the text to mark the low bound (50th percentile GM distribution) and high bound (5th percentile CSF distribution). The T2w/T1w signal is binned according to the low and high bound and assigned a TIGR score within the lesion mask. Pink outline, image in native space; green outline, image in MNI space.

The workflow of calculating the TIGR maps is depicted graphically in Figure 1. The user input includes a T1w and T2w image as well as a binary lesion mask in native space. First, high-resolution T1w and T2w images are denoised with an optimized non-local means filter^1^ (Coupe et al., 2008; Wiest-Daessle et al., 2008) to remove Rician noise from magnitude images (Gudbjartsson and Patz, 1995). Removing Rician noise from both the T1w and T2w images improves the signal-to-noise ratio and therefore improves the subsequent division of the two signals (see Supplementary Section “Logic of Denoising Images as Initial Processing Step” for further details).

The denoised T1w and T2w images are then co-registered together *via* FreeSurfer’s boundary-based registration (bbregister) (Greve and Fischl, 2009), resulting in good alignment between the two modalities in preparation to divide the T2w image by the T1w image on a voxel-wise basis. Each type of image (T1w and T2w) encodes unique signal information of the underlying tissue morphology. Taking the T2w/T1w ratio combines both types of information into one image to highlight the gradient of tissue damage within the lesion (see Supplementary Figure 1 and accompanying text for more details as to why T2w/T1w ratio was chosen instead of T1w/T2w). To compare TIGR maps and functional maps across subjects and generate brain–behavior relationships, the T2w/T1w ratio maps are spatially normalized to MNI template space (see Supplementary Figure 2 and accompanying text for more details on the “chimera spatnorm pipeline”).

To scale the T2w/T1w signals to a subject-specific value that can be compared across the entire cohort, the signal intensity is bounded by gray matter (GM; low bound: 0.1) and cerebrospinal fluid (CSF; high bound: 1.0) and classified into nine “bins” between 0.1 and 1.0 to maintain comparability with binary lesion maps that are characterized by zeros and ones. The computation of the thresholds and bins is described in the following paragraphs.

The high bound (CSF) threshold is obtained as follows: segment the contra-lesional lateral ventricle (Patenaude et al., 2011) and further restrict the signal to the anterior portion to avoid the choroid plexus (*y* > 15 in MNI space). The contra-lesional anterior lateral ventricle is then eroded by one voxel to avoid partial voluming with caudate GM and corpus callosum white matter (WM). Finally, the T2w/T1w signal of the eroded contra-lesional anterior ventricle mask is plotted in a histogram and empirically thresholded at the fifth percentile. Anything at or greater than this “high bound” T2w/T1w signal threshold is assigned a value of 1.0 and denoted as “most damaged.”

The low bound (GM) threshold is obtained as follows: the gray matter ribbon is segmented (Zhang et al., 2001) and further restricted to the contra-lesional anterior portion (*y* > 15 mm) to maintain similar signal restrictions to the ventricle segmentation. As can be seen in Supplementary Figure 3, the anterior contra-lesional hemisphere GM ribbon includes the frontal lobe, anterior subcortical areas such as the caudate head, and the anterior temporal pole. To avoid partial voluming with adjacent WM, the contra-lesional anterior GM ribbon is eroded by one voxel. Finally, the T2w/T1w signal under the eroded contra-lesional anterior GM ribbon is plotted in a histogram, and the 50th percentile is denoted as the low bound and assigned a value of 0.1. Any T2w/T1w signal below this “low bound” threshold is assigned a value of 0 and denoted as “healthy.” The 50th percentile of the GM signal was empirically chosen as the low bound *via* inspection of the images, histograms, and resulting TIGR maps.

**FIGURE 2:**
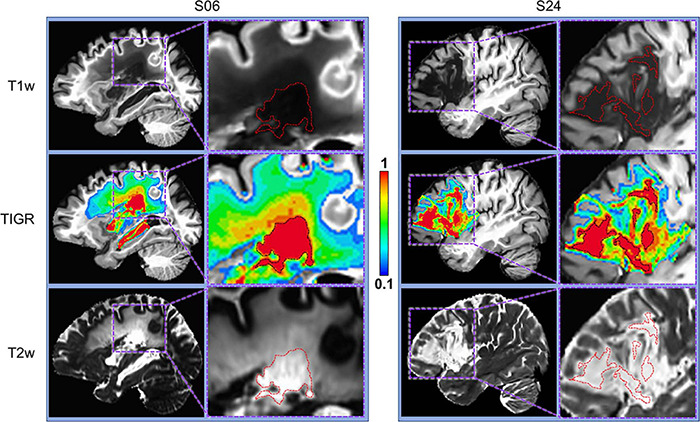
Comparison of T1w, T2w, and TIGR maps from two chronic stroke participants. Each participant has a distinct lesion phenotype, including lesion location (S06, subcortical; S24, frontal) and characterization of lesion core and pericavitational perimeter. The dotted red or black lines in the zoomed images denote the cavitation, showing that identifying lesion core and quantifying the gradient in tissue damage from either T1w or T2w image alone may be difficult.

To obtain the scaled TIGR scores, the T2w/T1w signal intensities are binned in a linear fashion between the low bound (0.1) and the high bound (1.0) in nine steps from “least damaged” to “most damaged.” Only the voxels within the user-defined lesion mask are classified into the tissue gradient “bins,” creating the final TIGR map used in group analysis. The color bar in Figures 1, 2 ranges from blue (0.1, low damage) to red (1.0, high damage) and estimates the degree of tissue damage as quantified by the TIGR map in the lesioned area. We ultimately plan to make our pipeline publicly available for use by others.

### pCASL MRI Acquisition and CBF Processing

Of the 35 subjects with TIGR scores, pseudo-continuous arterial spin labeling (pCASL) MRI was collected in a subset of participants (*N* = 6) to compute regional CBF maps. The pCASL method acquires control and label images, the subtraction of which yields pure blood signal that is directly proportional to CBF and can be mapped on a voxel-wise basis to obtain regional blood flow information. The subjects are indicated in Supplementary Table 1 with a “Y” in the CBF map column.

The pCASL images were collected with the following sequence parameters on Siemens 3T Prisma: 2D ascending gradient echo planar imaging (EPI) acquisition, 35 slices, slice thickness = 4 mm, 10% gap, matrix = 74 × 74, field of view (FOV) = 220 mm × 220 mm, in-plane resolution = 3 mm× 3 mm, GRAPPA = 2, no partial Fourier, acquisition bandwidth = 2,505 Hz, TR = 5,060 ms, TE = 13 ms, postlabeling delay = 2,200 ms, labeling duration = 1,500 ms, label offset = 90 mm. An additional M0 scan was acquired with the same brain coverage as the pCASL scan, except for a longer repetition time (TR = 10 s) to allow for fully relaxed magnetization to remove proton density effects during CBF quantification.

The analysis of pCASL data is accomplished with in-house scripts using a combination of AFNI and FSL commands. First, bulk-head motion correction is computed with AFNI’s 3dWarpDrive using six degrees of freedom. The motion parameters are used to censor pairs of label and control images that contain motion of >0.5 mm and >5° of rotation. A minimum of 30 pairs were used for every participant’s dataset. The label and control images were sorted, smoothed by 5 mm, then subtracted in native space to obtain the difference signal (=control − label), and then averaged prior to conversion to physiological units. To obtain CBF in physiological units, the difference signal (=control − label) and M0 image are combined with a single-compartment model to obtain units of ml/100g/min (Buxton et al., 1998). The CBF map was then transformed into MNI space by co-registering to T1w space using FSL’s flirt and subsequently applying the chimera warp into MNI space.

### Validation of TIGR-Identified Pericavitational Regions Using CBF Maps

**FIGURE 3:**
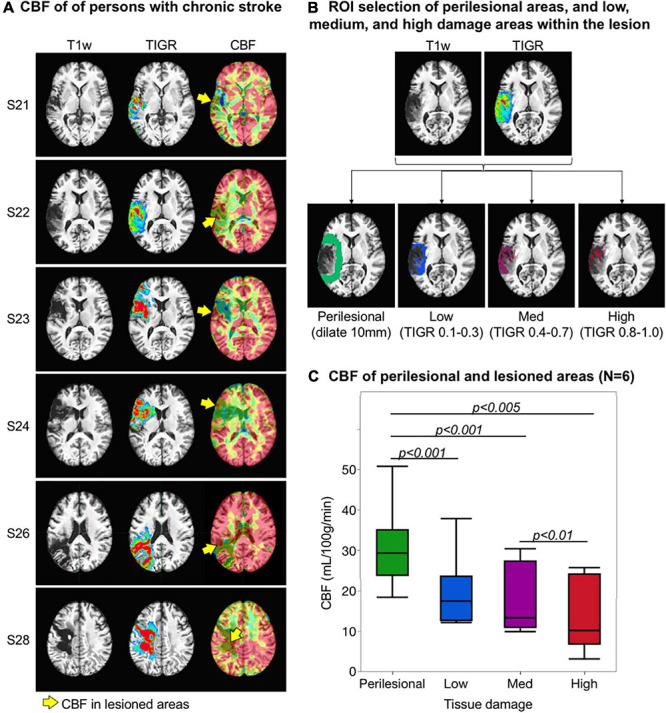
Validation of tissue integrity gradation *via* T2w T1w ratio (TIGR) using cerebral blood flow (CBF) maps. **(A)** The T1w image, TIGR map, and corresponding CBF map from six chronic stroke participants. The yellow arrows highlight the perfused pericavitational areas. **(B)** The region of interest selection of a representative individual (see text for description). **(C)** Pericavitational areas present with a significantly lower blood flow than the perilesional areas, where high damage areas have the lowest quantified CBF.

To validate TIGR, we set out to test if the amount of tissue damage quantified by TIGR could explain the amount of regional cerebral blood flow. Tissue damage was categorized into four regions of interest (ROIs): perilesional area and low-, medium-, and high-damage lesioned areas. The perilesional mask was generated by dilating the individual’s lesion map by 10 mm and subtracting the lesion area (Geranmayeh et al., 2015). The low-damage mask was generated by combining all voxels with TIGR score 0.1 to 0.3. The medium-damage mask was generated with TIGR scores 0.4 to 0.7, and the high-damage map was generated with TIGR scores 0.8 to 1. These masks are unique to each subject based on their lesion location, TIGR profile, and size. A representative set of ROIs is shown in Figure 3B. For each participant with CBF maps, an average CBF value was computed for each ROI. We completed an ANOVA in JMP Pro15 (Cary, NC, United States) to test if tissue damage explains the amount of regional cerebral blood flow. The results of the model are reported with *F*-statistic, and subsequently two-tailed paired *t*-tests are performed to determine which terms had a significant effect.

### Task fMRI Acquisition and Processing

Of the 35 subjects with TIGR scores, a subset (*N* = 14) participated in an overt language task fMRI scan collected on the Philips Achieva system. The subjects are indicated in Supplementary Table 1 with a “Y” in the task fMRI column. The event-related overt task fMRI paradigm for semantic category member generation has been successfully used by our group in persons with aphasia (Benjamin et al., 2014; Krishnamurthy et al., 2021). The participants were shown a category on a screen (e.g., tool, fruit, and farm animal) to which they were instructed to overtly generate a member of that category (e.g., hammer, apple, and horse). The task fMRI protocol was acquired using a single-shot gradient recalled EPI sequence with the following parameters: 36 sagittal slices, slice thickness = 4 mm, FOV = 240 mm × 240 mm, matrix size = 64 × 64, TR = 1,700 ms, TE = 30 ms, FA = 70°, acquisition bandwidth = 3,906 Hz/px, 186 volumes per run, and a total of six runs. The fMRI image processing involved (i) slice time correction to account for timing offset between acquired slices, (ii) rigid body motion correction *via* volume registration across all six runs, and (iii) followed by spatio-temporal independent components analysis (ICA) denoising for non-rigid speech-induced task-correlated motion (Krishnamurthy et al., 2021). Note that the ICA denoising classifier also incorporated correction for physiological noise, hardware artifacts, motion-related rimming artifacts, and susceptibility-motion artifacts. Next, (iv) we employed FSL-based epi_reg and FreeSurfer-based boundary-based registration tools to obtain a well-aligned co-registration between MPRAGE and task fMRI images. In conjunction, the MPRAGE was spatially normalized to MNI space with the chimera pipeline as described above, (v) The byproduct of this step (i.e., the warp image) was applied on the denoised and co-registered task fMRI images, (vi) We also developed a brain mask with the lateral ventricles removed to guide the spatial smoothing (with a kernel of 6 mm) to minimize CSF dilution of task activity in subcortical structures, (vii) The smoothed BOLD time course was scaled with respect to the initial baseline to obtain relative percent of BOLD change (Krishnamurthy et al., 2021), censored for head motion (>0.3 mm), followed by deconvolution (3dDeconvolve of AFNI) of the combined 60 trials across all six runs. To minimize low-frequency scanner drifts, we utilized polynomial fitting within the 3dDeconvolve command of AFNI. The deconvolution step also incorporated regressors for the different motion parameter estimates and its derivatives obtained *via* 3dvolreg and 1d_tool.py AFNI commands, and (viii) Finally, the statistical parametric activation maps on each individual participant were generated at statistical threshold *F* > 4.9 (*p* < 1.0 × 10^–7^, cluster size = 2 voxels). The relatively less stringent cluster thresholding for multiple comparison was deemed as optimal because the residual activated tissue/voxels within the lesion is expected to be few and variable across participants.

### Validation of TIGR-Identified Pericavitational Regions Using Task fMRI

**FIGURE 4:**
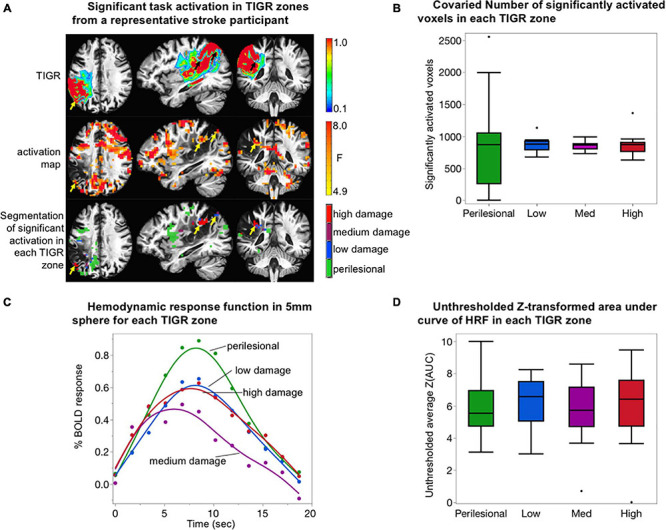
Validation of tissue integrity gradation *via* T2w T1w ratio (TIGR) using task fMRI. **(A)** The TIGR maps (top row) and thresholded activation maps (middle row) of a representative participant (S03). The combination of these two modalities helps identify functional pericavitational areas with low, medium, and high damage. Perilesional areas with a significant task activity were identified with yellow/black arrows. **(B)** The co-varied number of significantly activated voxels in each TIGR zone for *N* = 14 participants. No difference in activation volume between tissue damage zones was detected. **(C)** Average HRF curves for *N* = 14 subjects in perilesional (green), low-damage (blue), medium-damage (purple), and high-damage (red) areas. The HRF curve for medium-damage areas is significantly lower than the other three tissue types. **(D)** Plot representing BOLD energetics in each tissue damage zone. No difference in BOLD energetics between tissue damage zones was detected.

Task fMRI serves as a marker of brain regions engaged during the task and is therefore used to further validate TIGR and its ability to identify pericavitational areas. First, the perilesional areas and low-, medium-, and high-damage lesioned areas are generated for each individual subject as described in the CBF section, except that each ROI is resampled to EPI acquisition resolution. Then, the thresholded task activation is further constrained into the four respective ROIs (Figure 4A).

The number of activated voxels, as a function of tissue viability, was assessed by counting the number of significantly activated voxels in each TIGR zone (perilesional, low damage, medium damage, or high damage), followed by co-varying the total number of voxels in each zone to remove the effects of tissue volume. The co-varied number of significantly activated voxels was then entered into an ANOVA in JMP Pro15 to determine if tissue viability explains the number of activated voxels in each zone. The results of the model are reported with *F*-statistic.

To assess if task activity of pericavitational areas is physiologically meaningful, the hemodynamic response function (HRF) was extracted to inspect its shape. To extract the HRF from each participant, a 5-mm sphere (seven voxels) was drawn around the voxel with the highest *F*-statistic within a TIGR zone and averaged. The HRFs were then group-averaged irrespective of lesion location. The data was plotted in JMP Pro15, and differences between HRFs from different tissue zones were compared using paired *t*-test.

Finally, to assess if the BOLD energetics changed as a function of tissue viability, we extracted the unthresholded Z-transformed area under the curve of the HRF (Krishnamurthy et al., 2020, 2021). Briefly, we computed the area under the curve (AUC) of all beta coefficients describing the HRF (12 tent functions that jointly describe the 3dDeconvolve output impulse response function), followed by a Z-transform of the AUC for each participant to normalize the distribution in preparation for parametric statistics. The Z(AUC) was then averaged across perilesional and low-, medium-, and high-damage zones without statistically thresholding the number of voxels. Although thresholding is important to interpret functional activity, we decided not to threshold the BOLD energetics because thresholding can bias which networks are detected on an individual basis (Wilson et al., 2017). To ensure that the size of each damage zone does not influence the comparison of BOLD energetics across each zone, we co-varied the number of voxels in each zone from the average Z(AUC) prior to performing an ANOVA in JMP Pro15 to inspect if Z(AUC) was dependent on the amount of tissue damage.

### Multivariate Brain–Behavior Relationships

We assessed the feasibility of quantifying brain–behavior relationships with WAB language behavior and TIGR using the sparse canonical correlation analysis (sccan) of LESYMAP thresholded at *p* < 0.05 and false discovery rate (FDR)-corrected (Pustina et al., 2018). To assess if TIGR is able to capture reasonable brain–behavior results, we show the results for four imaging modalities: binary lesion maps, TIGR maps, metrically scaled T1w images (T1w-only), and metrically scaled T2w images (T2w-only), all in MNI template space to facilitate group statistics and comparison across methodologies. There were no covariates such as lesion size or age entered into the modeling of the brain–behavior relationship for any of the tested modalities. The lesion maps for the 35 participants are the same as used to quantify TIGR: extracted with LINDA in native space and then spatially transformed to MNI space with the chimera transform as described previously.

### Feasibility of TIGR in Early and Late Subacute Stroke

Tissue integrity gradation *via* T2w T1w ratio MRI at three post-stroke phases (early subacute, late subacute, and chronic) was assessed on two participants (58 years old male and 60 years old male) obtained from a publicly available stroke dataset (Corbetta et al., 2015; Siegel et al., 2017). The T1w and T2w images were assessed for motion and then processed to quantify TIGR as described previously. The lesion mask was computed using LINDA on the chronic dataset and applied to all three timepoints to impose consistency in areas in which TIGR MRI was evaluated. The chronic timepoint was chosen for the lesion quantification because LINDA is optimized for chronic stroke lesions. The number of voxels for each TIGR score bin was extracted for each post-stroke phase. Because the number of quantified TIGR voxels varied by post-stroke phase (despite using the same lesion mask), we co-varied the total number of voxels from the number of voxels in each TIGR score bin. The co-varied number of lesion voxels were then entered into an ANOVA in JMP Pro15 to determine if TIGR score, post-stroke phase, and the interaction between TIGR score and post-stroke phase explain the variability in the TIGR score from one timepoint to another. The results of the model are reported with *F*-statistic and the parameter estimates with *t*-statistic.

## Results

### Identification of Pericavitational Areas Using TIGR

As depicted in Figure 2 and Supplementary Figure 1, T1w and T2w images from chronic stroke survivors each contribute unique information to determine tissue composition changes based on voxel intensity values. When combined, the T2w/T1w ratio and subsequent TIGR map incorporate the signal contrast from both modalities, providing a delineation of the cavitation (red) and pericavitational perimeter (blue to orange) as well as the gradient of tissue damage across the span of the lesion.

### Validation of TIGR-Identified Pericavitational Areas Using CBF Maps

Cerebral blood flow maps for six chronic stroke participants are shown in Figure 3A alongside their respective TIGR maps and T1w images. The CBF values across all participants were in the expected physiological range with good separation of GM and WM blood flow, except in areas of lesion. As a first evidence that damaged tissues may still be viable, select lesioned areas do show residual blood flow (Figure 3A, yellow arrows). To quantify the amount of CBF in perilesional and lesioned areas, subject-specific ROIs were computed based on their lesion and TIGR maps (see Figure 3B for the ROIs of a representative participant). The CBF was then averaged for perilesional areas as well as low-, medium-, and high-damage areas within the lesion to determine a pattern of blood flow reduction based on tissue damage (Figure 3C). The ANOVA revealed a significant relationship between tissue damage and regional CBF [*F*(1,22) = 9.69, *p* = 0.005]. Subsequent two-tailed paired *t*-tests revealed a significant difference between perilesional and low-damage areas [*t*(5) = −7.72, *p* = 0.0006], perilesional and medium-damage areas [*t*(5) = −4.07, *p* = 0.0097], perilesional and high-damage areas [*t*(5) = −5.13, *p* = 0.0037], and medium- and high-damage areas [*t*(5) = −4.20, *p* = 0.0085]. There was no difference between low- and medium-damage areas and only a trending difference between low- and high-damage areas. To inspect if TIGR provides unique information compared to LINDA lesion probability maps, please see the Supplementary Section “LINDA Lesion Probability Maps Compared to TIGR Maps and CBF Relationship.”

### Validation of TIGR-Identified Pericavitational Areas Using Task fMRI

To further validate TIGR, we tested if task activity could be identified in pericavitational areas in *N* = 14 participants. As observed in Figure 4A and Supplementary Figures 5–9, a robust and physiologically meaningful task activity generally does occur inside the lesion, but in smaller clusters due to the reduced non-cavitated tissue volume available (yellow and black arrows in Figure 4A). The number of significantly activated voxels was counted in each TIGR zone and then co-varied with ROI size to remove the effects of tissue volume. There is no effect of tissue damage zone on the number of significantly activated voxels when the ROI size is taken into account (Figure 4B) as tested with an ANOVA [*F*(1,54) = 0.0003, *p* = 0.98].

The evaluation of the HRF from areas adjacent and within the lesion shows a gamma function characteristic of a BOLD response from perilesional areas (green curve, Figure 4C), low-damage areas (blue curve, Figure 4C), medium-damage areas (purple curve, Figure 4C), and high-damage areas (red curve, Figure 4C). The perilesional HRF displays the highest percent change in BOLD amplitude, followed by low-damage and high-damage areas and then medium-damage areas. Using a two-tailed paired *t*-test, we tested for significant differences between the curves. We found no differences between perilesional and low-damage and high-damage areas (*t* < 1.77, *p* > 0.08). However, medium-damage areas had a significantly lower HRF from the other three tissue areas (*t* > 4.31, *p* < 0.0001). These analyses were only carried out for the evaluation of TIGR MRI because it is unclear where the cavitation and pericavitational boundaries should be defined for T1w and T2w images.

Given the differences in the HRF curves, we quantified the unthresholded Z-transformed area under the curve of the HRF to further inspect if differences in BOLD energetics could be identified across different tissue damage zones. As can be seen in Figure 4D, the average unthresholded Z(AUC) does not depend on tissue viability as tested with ANOVA [*F*(1,54) = 0.0115, *p* = 0.91].

### Multivariate Brain–Behavior Results

**FIGURE 5:**
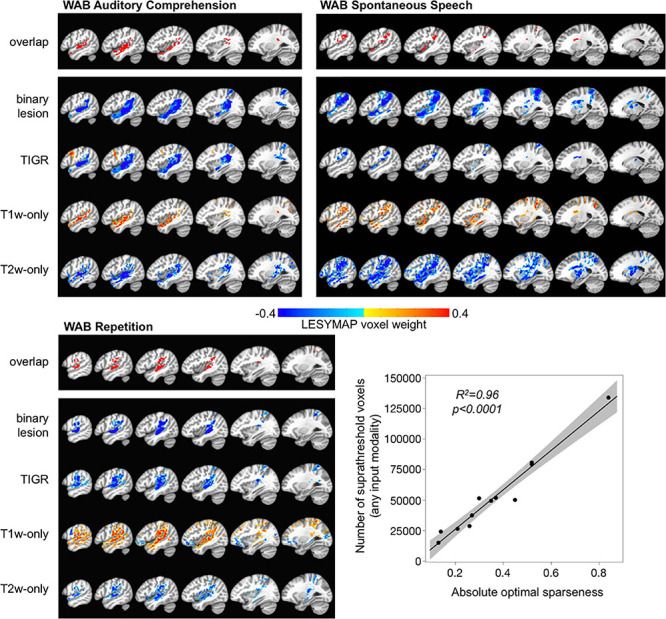
Multivariate brain–behavior relationships for WAB auditory comprehension, WAB repetition, and WAB spontaneous speech using binary lesion, tissue integrity gradation *via* T2w T1w ratio, T1w-only, and T2w-only maps. The overlapping map across the top for each behavior shows which brain areas were identified across all modalities for comparison purposes. The color bar represents LESYMAP voxel weights. The location of brain–behavior maps is similar across all modalities, but the extent of the maps differs based on the LESYMAP internally computed optimal sparseness (regression plot).

To demonstrate the feasibility of using TIGR maps to extract brain–behavior relationships, we used a multivariate analysis in *N* = 35 participants that relate all voxels simultaneously to WAB language behavior collected outside of the scanner with a model using sparse canonical correlations as implemented in LESYMAP (Pustina et al., 2018). The brain–behavior analysis for WAB Spontaneous Speech, WAB Repetition, and WAB Auditory Comprehension were significant (*p* = 0.05, FDR-corrected) and unique to the tested language behavior. These results are shown in Figure 5. The brain–behavior analysis was performed for four imaging modalities—binary lesion masks (Bates et al., 2003), TIGR maps, T1w-only (Tyler et al., 2005), and T2w-only imaging modalities—and are displayed together to facilitate comparison. Furthermore, an overlap map representing brain areas from all imaging modalities is generated to pinpoint nodes within the language network identified by all four modalities but does not represent the ground truth and is only displayed for the purpose of comparison. The cluster sizes, location, and represented brain areas are documented in Supplementary Tables 2–4.

The LESYMAP voxel weight in Figure 5 represents the contribution of the voxel to the entire model because all voxels are entered into the model simultaneously. The binary lesion maps, TIGR, and T2w-only maps show “blue” LESYMAP voxel weights because a larger signal intensity value represents more damage and is related to a lower behavioral score (negative slope). The T1w-only maps show “orange” LESYMAP voxel weights because a smaller signal intensity value represents more damage and is related to a lower behavioral score (positive slope). Thus, it is feasible to use TIGR as a continuous tissue damage metric and relate to behavior to extract brain–behavior relationships, indicating that TIGR tissue classification can be compared across participants.

Of note in Figure 5 is that the TIGR brain–behavior results are similar in extent across binary lesion maps and TIGR maps for WAB auditory comprehension and WAB repetition but are more thinly distributed for TIGR in WAB spontaneous speech. To explore this, we looked further at the LESYMAP output. We found that the number of suprathreshold voxels that LESYMAP creates does not depend on its internally run cross-validation correlation [*F*(1,10) = 2.01, *p* = 0.19] or the output cross-validation *p*-value [*F*(1,10) = 0.08, *p* = 0.78]. Instead the number of voxels in the statistical output map depends on the optimal sparseness computed within the LESYMAP algorithm [Figure 5 linear regression plot, *F*(1,10) = 227.72, *p* < 0.0001].

### Feasibility of TIGR in Early and Late Subacute Stroke

**FIGURE 6:**
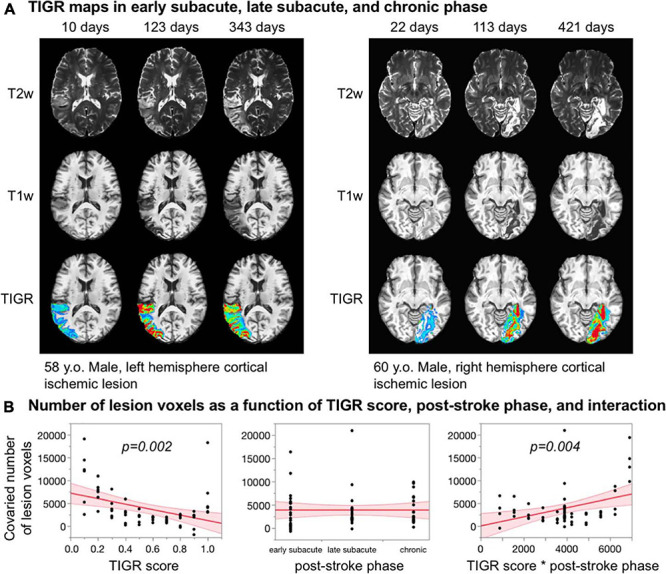
**(A)** Feasibility of quantifying tissue integrity gradation *via* T2w T1w ratio (TIGR) in early subacute, late subacute, and chronic phases post-stroke in two participants. **(B)** The number of voxels in each TIGR bin decreases with increasing TIGR score, does not relate with post-stroke phase, but depends significantly on the interaction between TIGR and post-stroke phase.

As can be seen in Figure 6A, TIGR can be quantified in early subacute, late subacute, and chronic phases post-stroke. The underlying cellular composition changes across these three phases influences the tissue T2 and T1 and, in turn, causes the signal intensity of the T1w and T2w images to change. The T2w lesion signal intensity transitions from bright to brighter during each of the three phases for both participants. The T1w lesion signal intensity is initially insensitive to detecting tissue damage but, with time, becomes darker. Visually, this transitional effect can be seen in the TIGR maps. To determine if the transition of TIGR score is significant, we quantified the number of lesion voxels in each TIGR bin for every session and co-varied the total number of lesioned voxels to remove the effect of tissue volume. The ANOVA revealed a significant relationship for the whole model [*F*(3,56) = 6.74, *p* = 0.0006]. The parameter estimates (Figure 6B) showed that TIGR score had a significant negative relationship with co-varied number of lesion voxels (indicating fewer lesioned voxels in the higher-damage areas, *t* = −3.33, *p* = 0.002), no effect of post-stroke phase (because the total number of lesioned voxels was co-varied out, *t* = 0.0, *p* = 1.0), and a significant effect of interaction between TIGR score and post-stroke phase (indicating that lesioned voxels transition from lower to higher TIGR scores with increasing time post-stroke, *t* = 3.03, *p* = 0.004). These results on two participants of ischemic lesions are preliminary and must be replicated further in the literature.

## Discussion

We have developed a novel methodology, called TIGR, to objectively and quantitatively identify pericavitational areas and map gradients of tissue damage within the chronic stroke lesion. We validated the presence of viable tissue within the lesion by showing that both blood flow and functional task activity can be detected in TIGR-identified pericavitational areas. We also showed the feasibility of quantifying brain–behavior maps using TIGR, indicating that TIGR maps can likely be compared across participants and scanners because they are individually normalized to subject-specific GM (low bound) and CSF (high bound) thresholds. Finally, we demonstrated the feasibility of quantifying TIGR in early and late subacute stroke and that TIGR identifies a transition of tissue damage from early subacute to chronic post-stroke phases.

By labeling the TIGR maps from “least” to “most” damaged, we have hypothesized that the false color scale is indicative of tissue damage. Although we have non-invasively validated TIGR with CBF and task fMRI to ultimately test this hypothesis, future studies will have to compare the histology and TIGR MRI in animal models to determine how morphological cellular changes correspond to the TIGR classification of tissue damage, for example, reactive astrocytes swell and have partially overlapping endfeet close to the glial scar but reduce in size further from the lesion core, gradually becoming healthier until indistinguishable from normal astrocytes (Burda and Sofroniew, 2014). The number of reactive astrocytes is related to the hypoxic stress experienced by brain tissue during stroke (Anderson et al., 2014) and is likely related to the amount of collateral flow available to the brain area. TIGR maps generally show a reduction in TIGR values toward the periphery of the lesion (i.e., less damage, blue), where fewer reactive astrocytes and more surviving neural-lineage cells are expected to be found. Histology-imaging comparisons in animal models will determine how faithfully TIGR maps report on underlying cellular changes after stroke and may pose an opportunity to further optimize the TIGR GM and CSF thresholding limits, including the number of bins required to describe the gradation of tissue damage. The choice of GM and CSF thresholds at this point is subjective until further work is done to compare the TIGR maps to histological metrics of neuronal and glial morphological changes in lesioned tissue. Adjusting the threshold limits will change the assignment of TIGR score to the T2w/T1w signal intensity, which will change the classification of tissue damage. We acknowledge that until imaging–histology comparisons are carried out, the subjectivity of the GM and CSF thresholding is a limitation of the TIGR methodology.

We have validated the notion that pericavitational areas may contain viable tissue by showing that CBF can be detected in these damaged regions. We then validated the classification of tissue damage of TIGR by quantitatively relating the amount of blood flow in absolute physiologic units, which is a surrogate for metabolic demand (Drake and Iadecola, 2007), to the degree of tissue damage, where low-damage areas have a greater blood flow than medium- or high-damage areas but is significantly reduced compared to that in adjacent perilesional areas. The assumption here is that the metabolic demand and supply is greater for less damaged tissues. It is known that the lesioned and adjacent areas have compromised vascular properties such as maximally dilated vessels supporting damaged tissues (Krainik et al., 2005; Papassin et al., 2020). Although blood flow is lower in pericavitational areas, we expect the cerebrovascular reactivity (CVR) to also be lower due to a maximally dilated vasculature (Juttukonda and Donahue, 2019). To further understand the vascular properties and rehabilitation potential of pericavitational areas, it will be important to investigate the TIGR–CBF relationship in a larger cohort and to explore other underlying vascular properties, such as CVR within the pericavitational areas.

Our use of detecting meaningful task fMRI activation in TIGR-identified pericavitational areas helped to further validate TIGR MRI. Task activation was chosen as a means of validating TIGR because (1) CBF maps can identify which regions within the lesion are metabolically active but do not necessarily indicate functional areas and (2) areas of significant task activity are important to define domain-specific regions that can be targeted during rehabilitation. Task activation was observed at TIGR thresholds between 0.1 and 0.9 (with some inclusion of cavitation edge voxels), which could further inform the level of tissue compositional changes that may facilitate successful rehabilitation. It was notable that the HRF amplitude was lowest for medium-damage tissue areas, while the low-damage and high-damage areas peaked at a higher % BOLD change. However, this is not a function of the number of significantly activated voxels in each TIGR zone, as there is no difference after accounting for the number of voxels in each zone. The HRF is indicative of concomitant metabolic and vascular processes in response to stimuli (Buxton et al., 2014), but it is unclear why medium-damage areas have a lower peak than the other zones and thus require additional work and reproducibility of our findings. We also found that the Z-transformed area under the curve of the HRF is not dependent on tissue damage. This is not a negative finding as AUC is agnostic to HRF parameters (i.e., time-to-peak and peak amplitude). While individual HRF parameters can be sensitive to vascular changes, the AUC is reflective of the task-induced BOLD energetics. Thus, although the HRF shape seems to change across tissue damage due to underlying vascular differences (Figure 4C), the cumulative BOLD energetics (described by AUC) does not. There could be multiple factors at play that could cause the AUC to remain constant across tissue damage even though the baseline CBF is reduced with increasing tissue damage, but most likely this points to a difference in basal cerebral metabolic rate of oxygen (CMRO_2_) and activity-driven CMRO_2_ change across tissue damage types (Ibaraki et al., 2004). Future studies examining task fMRI in stroke, especially perilesional and lesioned areas, should consider neuro-sensitizing the task fMRI signals for variations in regional CBF (Krishnamurthy et al., 2020) such that the interpretation is specific to the neural side of the equation. One limitation of our task fMRI study is that, for this previously collected task fMRI dataset, our group did not collect in-scanner task performance for accuracy. It is reasonable to expect that the subjects may miss some trials that can dilute the deconvolution estimates, but we have observed robust task-related activation in our previous work (Benjamin et al., 2014; Krishnamurthy et al., 2021) and a robust Fano Factor which is a measure of trial-by-trial variability on this dataset (Krishnamurthy et al., 2021). It will be interesting to characterize, in a future work, how neuro-sensitized task activation from pericavitational areas relates to task performance and may further help to validate TIGR MRI. Another limitation of using task fMRI to validate TIGR is that the detection of task activity is not expected throughout the entire middle cerebral artery (MCA) territory and depends on factors beyond tissue damage, such as aphasia severity and task difficulty. In the future, task performance, lesion location, and other covariates should be taken into account when attempting to detect functional activation in damaged tissues.

The TIGR methodology was developed on chronic MCA territory lesions. Therefore, it was important to explore the ability of TIGR to quantify tissue damage in early and late subacute stroke phases, when rehabilitation potential is at its greatest. As a proof of concept, in two participants, we showed that TIGR can indeed be quantified in early subacute (as early as 10 days post-stroke) and late subacute stroke phases. Tissue remodeling is rapid during this time frame of only a few months, which can be seen from the T1w and T2w image signal intensities accompanying the TIGR maps. Based on the preliminary results in Figure 6, application of TIGR in the chronic phase may perhaps be most sensitive to gradients of tissue damage. The utility of TIGR in earlier time frames, such as acute or early subacute, may need more optimization of anatomic MR scan parameters and TIGR methodology, such that TIGR can also be utilized in acute and subacute rehabilitation. It may also be interesting to determine in future studies if TIGR in early subacute stroke phase can predict tissue damage at later stroke phases. Although the same lesion mask was applied to each stroke phase, the number of non-zero TIGR values increased from early subacute to late subacute to chronic stroke phase in these two participants. It may be that these regions should be targeted during intervention to preserve as much tissue as possible and reduce collateral damage. Because TIGR was developed on MCA infarcts, we also showed that TIGR could be utilized in other vascular territories, such as the posterior cerebral artery territory (fusiform gyrus infarct in Figure 6), pontine infarcts, and cerebellar infarcts (see Supplementary Figure 10). Together with the feasibility in subacute stroke, the ability to quantify tissue damage in other vascular territories shows a good generalizability of TIGR to stroke cases beyond chronic MCA.

The choice of binning the T2w/T1w ratio from 0.1 to 1 rather than metrically scaling by division was a deliberate one, which was designed to avoid introducing inter-subject variability. The T2w/T1w ratio, T1w-only, or T2w-only signals must be binned or metrically scaled to compare across subjects because head size and head position within the MRI head coil differ across subjects and the RF amplifier characteristics can vary daily, leading to arbitrary MR signal intensities that cannot be compared across subjects unless binned or scaled. In our proposed TIGR methodology, we chose to bin the signals between two known values (GM and CSF) rather than metrically scaling by dividing each voxel by the whole brain mean intensity [the current standard method for scaling T1w-only images (Tyler et al., 2005)]. Metric scaling by division removes arbitrary inter-subject variations in MR signal intensities in a general fashion but is still biased by participant differences in ventricle and lesion size. Due to stroke and aging-related changes, there may be large brain areas with dark (T1w) or bright (T2w) signal intensities that are not consistent across all subjects but are inadvertently included when dividing by the whole-brain average. Thus, binning the signals between two known tissue types (GM and CSF) removes the bias introduced by lesion or atrophy that occurs when applying metric scaling *via* division.

The identification of pericavitational perimeter could provide valuable information for clinicians in the rehabilitation setting, where it may be important to identify viable tissue available to reaquire a latent skill (Crosson et al., 2017). Patterns of stroke recovery have shown that successful skill reacquisition is accompanied by a return of activation in the ipsi-lesional hemisphere (Binkofski and Seitz, 2004; Corbetta et al., 2005; Saur et al., 2006) and that task activation that relocalizes closest to its original area is most beneficial and not a function of task effort (Ward et al., 2004; Bonstrup et al., 2015). This may be due to the presence of neurons and unsevered axons within the pericavitational perimeter that are able to take on the tasks of the non-functional tissue, provided the lesion spares, at least to some degree, parts of the local network previously utilized during behavior (Di Pino et al., 2014). TIGR maps could also provide an important step forward in our ability to design individualized non-invasive brain stimulation montages to selectively upregulate or downregulate brain areas during rehabilitation. We compared the lesion probability maps extracted with LINDA to TIGR, and as expected, we also observed a significant inverse relationship between lesion probability and cerebral blood flow. This finding is consistent with the TIGR–CBF relationship (Figure 3). However, given that TIGR score is spatially more confined to areas of high lesion probability (Supplementary Figure 4A), TIGR provides clinically relevant additional information. Specifically, the clinical utility of TIGR is likely to come in the form of targeted treatment planning, such as rTMS localization or optimal placement of transcranial direct current stimulation (tDCS) electrodes. Furthermore, for seed-based resting state functional connectivity analysis, having such spatial specificity for residual tissue will facilitate the potential of such tissue in whole-brain functional connectivity evaluations. The spatial extent and quantitative evaluation of tissue damage could also be used in current modeling programs for the optimal design of tDCS in a lesioned brain because electrical current propagates differently in CSF-filled spaces compared to tissue (Bikson et al., 2012), and dielectric effects at tissue boundaries with conductivity differences influence electrical current propagation (Sadleir et al., 2010). Therefore, mapping the tissue composition-related changes across the lesion with TIGR could aide the use of non-invasive brain stimulation as an adjuvant to behavioral interventions, affording individualized neuromodulation in rehabilitation.

As a proof of principle, we demonstrated that TIGR maps can identify group-level brain–behavior relationships, where continuous values of behavior are statistically related to continuous TIGR scores in a multivariate fashion that considers all voxels simultaneously. Brain–behavior maps derived from TIGR are similar in location to binary lesion masks, which indicates that the TIGR tissue classifications can be used across participants and has relationship to behavior. However, the brain–behavior maps generated from binary lesion, TIGR, T1w-only, and T2w-only differed in extent, which we showed as relating to the internally computed LESYMAP sparseness coefficient. In order to compare the extent of brain–behavior maps across modalities, the sparseness coefficient would have to be equated, but that is currently not recommended by other experts in the field (Pustina et al., 2018). A difference in optimal sparseness between modalities likely indicates differences in signal sensitivity to respective behavior, which might be critical in diagnosis and treatment planning that requires additional future work. Therefore, in order to determine if the brain–behavior results of one modality are “superior” or “complementary” to another modality, it may be important to compare the ability of one modality to predict a cognitive model against the ability of another modality to predict the same cognitive model. Synthetic simulations may add further information in identifying which modality is representative of the ground truth, which is worthwhile to pursue in future studies. Furthermore, it is important to consider that TIGR and the other modalities are computed from structural images and that additional imaging types such as functional MRI, perfusion MRI, and diffusion MRI may aide in identifying viable pericavitational tissue and its influence on behavior (Halai et al., 2020; Ptak et al., 2020). Future studies should combine structural and neurophysiological information to approach brain–behavior relationships from a multi-modal perspective to better explain variability in behavioral data.

This current study has several limitations. Strokes can be classified into ischemic (occlusion of a vessel; ∼80% of cases) or hemorrhagic (rupture of a vessel, ∼20% of cases) strokes. One marked difference between these types of stroke is that the rupture of a vessel during a hemorrhage causes blood to spill into the tissue. During inflammatory processes and tissue remodeling stages, blood proteins and most cellular fragments due to hemorrhage are removed (Burda and Sofroniew, 2014), but the iron-containing hemosiderin stain remains even in the chronic stages of stroke. Upon inspection of the images and TIGR maps, we determined that the current implementation of TIGR is not sensitive to hemosiderin-stained areas. As a consequence, hemosiderin-stained areas from the cohort in this study may be misclassified as “healthy” tissue. The nine subjects in this study that presented with hemorrhages had prominent areas of ischemic damage, which justified their inclusion in the multivariate group analysis. The misclassification arises because the T2w signal for paramagnetic heme-containing tissues is small due to susceptibility-induced shortening of tissue T2. When the T2w/T1w ratio is computed for a heme-containing voxel, the ratio will be smaller than the GM (low bound) threshold and therefore not scored in the current TIGR classification scheme. Therefore, behavioral deficits that may be the result of hemorrhagic damage could be attributed to other lesioned areas from ischemia, leading to misinterpretation. Although the current implementation of TIGR can classify the tissue integrity of a majority of stroke cases (∼80%), future work should improve the acquisition or analysis of TIGR to classify damaged tissues with heme-containing compounds. Such optimization may include using MR acquisitions that are sensitive to iron-containing compounds, including static field maps, susceptibility-weighted imaging, quantitative susceptibility mapping, or R2^∗^ measures. Alternatively, it may also be possible to apply a separate thresholding system for tissues with a hemosiderin stain (by possibly using manganese-containing structures such as the putamen or deoxyhemoglobin-containing areas such as the superior sagittal sinus as reference) without acquiring additional scans. Another limitation stems from the HRF analysis. It is known that the BOLD response from different brain regions is distinct in shape, amplitude, and lag (Handwerker et al., 2004), but this report averaged across the HRF of all participants regardless of brain region (due to the heterogeneity of lesion location). Future studies may consider accounting for region of activity in the analysis of pericavitational activity to better understand the influence of tissue damage on the BOLD response. Another limitation is that this work combined data acquired on two different scanner systems. Due to the thresholding and analysis procedures, it is theoretically feasible to combine data across scanners, but this assumption must be validated in the future with “traveling head” studies. Finally, although our study uses a relatively small sample size of 35 subjects for brain–behavior analysis, we note that previous studies with similar sample sizes (Tyler et al., 2005) have successfully detected brain–behavior relationships. However, it would be of interest to investigate brain–behavior relationships with larger sample sizes (Rorden et al., 2007) by including TIGR MRI alongside binary lesion maps during brain–behavior quantification to determine if the continuous nature of TIGR provides more sensitive brain–behavior maps in larger cohorts.

## Conclusion

We have developed TIGR, a novel methodology that non-invasively classifies tissue composition changes within the lesion and can objectively identify pericavitational areas. This is a significant step to more accurately delineate the morphology of brain lesions for clinical applications. We have shown that these TIGR-identified pericavitational regions can be engaged during a task and result in meaningful HRF shapes. Furthermore, TIGR maps identify brain–behavior relationships on a similar level to binary lesion maps, indicating that TIGR tissue classifications can be compared across participants. Importantly, because the acquisition of high-resolution T1w and T2w scans is routine clinical practice, the implementation of TIGR maps to aid decision-making in rehabilitation can readily be implemented in clinical settings for a majority of stroke cases without additional imaging costs or patient burden.

## Data Availability Statement

The raw data supporting the conclusions of this article will be made available by the authors, without undue reservation.

## Ethics Statement

The studies involving human participants were reviewed and approved by the University of Florida, Gainsville and the joint review committee at Emory University and Atlanta Veterans Affairs Medical Center. The patients/participants provided their written informed consent to participate in this study.

## Author Contributions

LK, VK, and BC contributed to the conceptualization of the study and methodological development. BC contributed to the task design. LK, KM, and SH contributed to MR data collection. AR, GC, SH, and SB contributed to subject screening. LK, VK, CG, and SK contributed to data analysis. LK, VK, AR, KM, CG, GC, NR, SH, SB, SK, and BC contributed to the manuscript writing and editing. All authors contributed to the article and approved the submitted version.

## Conflict of Interest

The authors declare that the research was conducted in the absence of any commercial or financial relationships that could be construed as a potential conflict of interest.

## Publisher’s Note

All claims expressed in this article are solely those of the authors and do not necessarily represent those of their affiliated organizations, or those of the publisher, the editors and the reviewers. Any product that may be evaluated in this article, or claim that may be made by its manufacturer, is not guaranteed or endorsed by the publisher.

## Footnotes

1
https://sites.google.com/site/pierrickcoupe/softwares/denoising/mri-denoising
