# Supplementary material for: Not All Lesioned Tissue Is Equal: Identifying Pericavitational Areas in Chronic Stroke With Tissue Integrity Gradation via T2w T1w Ratio
Source: Front Neurosci. 2021 Aug 5;15:665707. doi: 10.3389/fnins.2021.665707 (PMC8378269; doi:10.3389/fnins.2021.665707)
Supplement: Supplementary file 1 [file Table_1.docx]

*Supplementary material for:*

**Not all lesioned tissue is equal: Identifying pericavitational areas in chronic stroke with Tissue Integrity Gradation via T2w T1w Ratio (TIGR)**

Lisa C. Krishnamurthy^*1,2^, Venkatagiri Krishnamurthy^1,3,4^, Amy D. Rodriguez^1,4^, Keith M. McGregor^1,4^, Clara N. Glassman^5^, Gabriell S. Champion^1^, Natalie Rocha^1^, Stacy M. Harnish^6^, Samir R. Belagaje^4,7^, Suprateek Kundu^8^, and Bruce A. Crosson^1,4,9^

^1^Center for Visual and Neurocognitive Rehabilitation, Atlanta VA Healthcare System, Decatur, GA, United States, ^2^Dept. of Physics & Astronomy, Georgia State University, Atlanta, GA, United States, ^3^Dept. of Medicine, Division of Geriatrics and Gerontology, Emory University, Atlanta, GA, United States, ^4^Dept. of Neurology, Emory University, Atlanta, GA, United States, ^5^Dept. of Nuclear and Radiological Engineering and Medical Physics, Georgia Institute of Technology, Atlanta, GA United States, ^6^Department of Speech and Hearing Science, The Ohio State University, Columbus, OH, United States, ^7^Department of Rehabilitation Medicine, Emory University, Atlanta, GA, United States, ^8^Department of Biostatistics & Bioinformatics, Emory University, Atlanta, GA, United States, ^9^Dept. of Psychology, Georgia State University, Atlanta, GA, United States

Running head: TIGR maps of pericavitational areas

Manuscript Key words: stroke lesion, TIGR, pericavitational area, task fMRI, CBF

**Supplementary Table 1**: Subject demographics, behavioral data, and lesion characteristics

| **Sub** | **Age** | **Sex** | **Mo. since stroke** | **Handed-ness** | **CVA type** | **WAB Spont. speech** | **WAB Auditory Comp.** | **WAB Repetition** | **WAB AQ** | **WAB Classi- fication** | **Lesion volume (mL)** | **Lesion location** | **Task fMRI** | **CBF map** |
| --- | --- | --- | --- | --- | --- | --- | --- | --- | --- | --- | --- | --- | --- | --- |
| S01 | 79 | F | 13 | R | H | 8 | 152 | 58 | 51.2 | Broca’s | 10 | Subcortical | Y |  |
| S02 | 52 | M | 98 | R | I | 16 | 150 | 58 | 74.4 | Conduction | 201 | Subcortical, Temporal |  |  |
| S03 | 92 | F | 30 | R | I | 12 | 183 | 56 | 69.1 | Conduction | 121 | Parietal | Y |  |
| S04 | 53 | M | 61 | R | I | 11 | 172 | 57 | 65.8 | Conduction | 232 | Parietal, Temporal | Y |  |
| S05 | 73 | M | 11 | R | I | 13 | 200 | 75 | 76.4 | Anomic | 59 | Insula | Y |  |
| S06 | 62 | F | 77 | R | H | 12 | 156 | 25 | 57.8 | Conduction | 161 | Subcortical | Y |  |
| S07 | 80 | F | 11 | R | I | 14 | 171 | 72 | 78 | Anomic | 65 | Insula, | Y |  |
| S08 | 55 | M | 10 | R | I | 10 | 179 | 90 | 52.2 | Transcortical Motor | 225 | Frontal, Parietal, Subcortical, Temporal | Y |  |
| S09 | 69 | M | 19 | R | I | 9 | 176 | 30 | 67.1 | Broca’s | 163 | Frontal, Parietal, Subcortical | Y |  |
| S10 | 68 | M | 207 | R | I | 14 | 161 | 40 | 67.8 | Conduction | 141 | Parietal, Temporal | Y |  |
| S11 | 63 | F | 12 | R | I | 16 | 172 | 44 | 66.6 | Conduction | 97 | Insula, Temporal | Y |  |
| S12 | 50 | M | 10 | R | I | 5 | 183 | 23 | 40.7 | Broca’s | 218 | Frontal, Parietal, Subcortical, Temporal |  |  |
| S13 | 61 | M | 11 | R | I | 13 | 184 | 78 | 75.8 | Anomic | 30 | Subcortical | Y |  |
| S14 | 68 | F | 18 | R | I | 11 | 188 | 62 | 69.4 | Broca’s | 168 | Parietal | Y |  |
| S15 | 59 | M | 37 | R | H | 12 | 169 | 91 | 74.5 | Anomic | 72 | Subcortical | Y |  |
| S16 | 42 | F | 8 | R | H | 15 | 183 | 80 | 82.5 | Anomic | 2 | Subcortical |  |  |
| S17 | 64 | M | 111 | R | I | 17 | 198 | 96 | 90 | Anomic | 84 | Insula | Y |  |
| S18 | 66 | M | 41 | R | I | 15 | 189 | 89 | 80.7 | Anomic | 135 | Frontal, Subcortical |  |  |
| S19 | 59 | M | 13 | R | I | 19 | 200 | 97 | 96.8 | Anomic | 65 | Frontal, Insula |  |  |
| S20 | 55 | M | 13 | R | I | 15 | 183 | 51 | 69.3 | Conduction | 35 | Subcortical, Temporal |  |  |
| **Sub** | ***Age*** | ***Sex*** | ***Mo. since stroke*** | ***Handed-ness*** | ***CVA type*** | ***WAB Spont. speech*** | ***WAB Auditory Comp.*** | ***WAB Repetition*** | ***WAB AQ*** | ***WAB Classi-fication*** | ***Lesion volume (mL)*** | ***Lesion location*** | ***Task fMRI*** | ***CBF map*** |
| S21 | 56 | M | 20 | R | I | 15 | 183 | 50 | 68 | Conduction | 60 | Insula, Temporal |  | Y |
| S22 | 35 | M | 9 | R | I | 4 | 124 | 24 | 27.4 | Broca’s | 159 | Insula, Temporal, Parietal |  | Y |
| S23 | 47 | M | 44 | R | H | 14 | 190 | 73 | 75.6 | Anomic | 152 | Frontal, Subcortical |  | Y |
| S24 | 43 | F | 49 | R | I | 14 | 190 | 86 | 80.6 | Anomic | 81 | Frontal, Subcortical |  | Y |
| S25 | 63 | M | 10 | R | H | 19 | 199 | 91 | 93.7 | Anomic | 1 | Subcortical |  |  |
| S26 | 81 | F | 60 | R | I | 14 | 161 | 67 | 74.1 | Conduction | 148 | Parietal, Temporal |  | Y |
| S27 | 80 | M | 43 | R | I | 19 | 190 | 100 | 94.8 | Anomic | 136 | Frontal, Subcortical |  |  |
| S28 | 73 | M | 55 | R | H | 14 | 178 | 88 | 79.6 | Anomic | 183 | Parietal |  | Y |
| S29 | 45 | M | 14 | R | I | 14 | 190 | 84 | 78.6 | Anomic | 167 | Frontal, Insula |  |  |
| S30 | 47 | M | 24 | R | I | 11 | 95 | 32 | 46.5 | Wernicke’s | 177 | Parietal, Temporal |  |  |
| S31 | 65 | F | 36 | R | I | 11 | 148 | 46 | 55 | Conduction | 120 | Insula, Temporal, Parietal |  |  |
| S32 | 45 | F | 84 | R | I | 10 | 138 | 28 | 52 | Broca’s | 363 | Frontal, Parietal, Subcortical, Temporal |  |  |
| S33 | 80 | F | 24 | R | H | 14 | 187 | 82 | 80.7 | Anomic | 19 | Subcortical |  |  |
| S34 | 61 | M | 132 | R | I | 10 | 97 | 32 | 43.5 | Wernicke’s | 255 | Subcortical, Temporal, Parietal |  |  |
| S35 | 52 | F | 60 | R | H | 16 | 174 | 96 | 84.4 | Anomic | 10 | Subcortical |  |  |

*Choice of T2w-divided-by-T1w versus T1w-divided-by-T2w and impact on damage gradient*


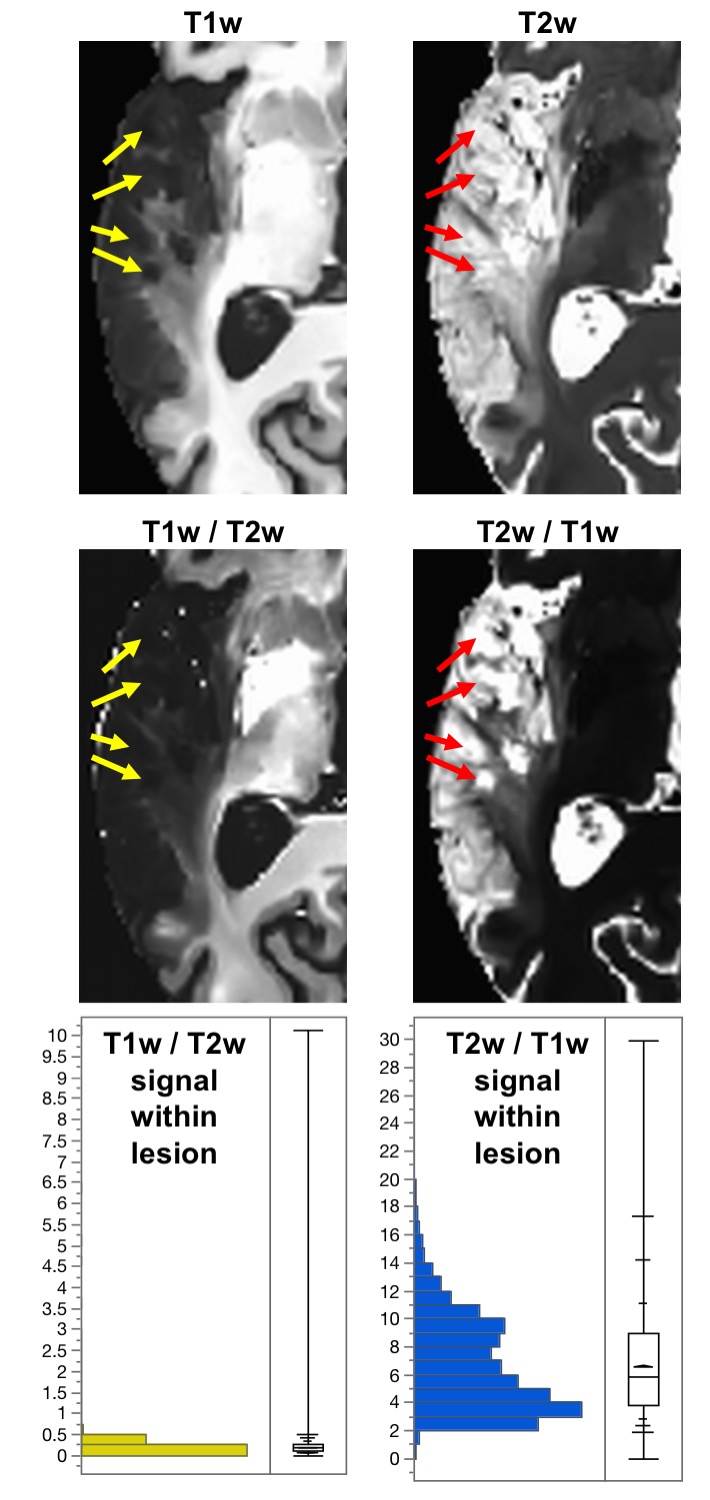


**Supplementary Figure 1:** A representative stroke participant’s (S22) T1w and T2w axial images of lesion and corresponding derived T1w/T2w and T2w/T1w images. The histograms represent the T1w/T2w (yellow) and T2w/T1w (blue) signal distributions within the lesion. The arrows denote high damage areas.

The user input for computing TIGR maps include a T1w and T2w image because each encodes unique information of the tissue morphology. The two images are combined via division. The choice of whether to divide T1w by T2w or vice versa is guided by the final divided signal intensity and its description of the tissue gradient.

TIGR is especially suited for ischemic lesions, where high damage areas are characterized by small T1w signal intensity close to zero, while T2w images in the same region have large signal intensity. Therefore,

S_T1w,lesion_ << S_T2w,lesion_ (SE1)

Where S_T1w,lesion_ is the T1w signal within the lesion and S_T2w,lesion_ is the corresponding T2w signal intensity. A representative participant’s lesion is shown in the top row of Supplementary Figure 1, where the yellow and red arrows point to example areas that meet the conditions of Equation SE1.

The second row of Supplementary Figure 1 shows the resulting signal intensities of T1w/T2w and T2w/T1w. The T1w/T2w signal intensities and contrast result from a number close to zero (T1w) that is divided by a large number (T2w), and therefore remains close to zero. Alternatively, for the T2w/T1w version, a large number (T2w) divided by a number close to zero (T1w) becomes very large. Inspecting the histograms and box plots on the third row of Supplementary Figure 1, the T1w/T2w signal distribution (yellow histogram) is narrow and thereby does not generate a robust damage gradient. On the other hand, the T2w/T1w signal distribution (blue histogram) has a much greater range that can be used to describe the tissue damage gradient.

*Logic of denoising images as initial processing step*

During the initial development of TIGR map analysis pipeline, early iterations did not include the denoising step, leaving small holes in the final TIGR map that had to be artificially smoothed with a Gaussian filter. Such signal blurring leads to local changes in the tissue integrity quantification, which could lead to misinterpretations of tissue damage. The denoising step also aids in improved segmentations and cross-modality coregistrations. Thus, we highly recommend the denoising of both T1w and T2w images as a first step in the processing of TIGR.

*“Chimeric brain” and MNI transformations in the presence of large cortical lesions*

The T2w/T1w ratio is spatially normalized with the “chimera spatnorm pipeline” (Supplementary Figure 2) to reduce the imperfections in the non-linear spatial transform from native space to MNI space due to signal intensity differences between the lesioned area and MNI template brain. The input to the chimera spatnorm pipeline includes a bias field corrected T1w image, a brain mask, and a lesion mask. First, the denoised T1w image is bias field corrected with FSL’s FAST(Zhang et al., 2001;Smith et al., 2004) (Supplementary Figure 2: image “1”) because intensity-based linear and non-linear estimations of spatial transforms are suboptimal in the presence of a bias field and can cause distortions in the final output. To skull strip the images, a binary brain mask (Supplementary Figure 2: image “2”) is generated using optiBET(Lutkenhoff et al., 2014), and manually touched up if necessary using ITK Snap to remove meninges and areas of calcification(Yushkevich et al., 2006). The estimation of an initial binary lesion mask (Supplementary Figure 2: image “3”) in native space is accomplished using LINDA(Pustina et al., 2016) and manually touched up with ITK Snap.

The chimera spatnorm pipeline is named for the fact that a “chimeric” brain is created by artificially stitching healthy tissue from the non-lesioned hemisphere into the lesion(Yourganov et al., 2018) to approximate the tissue geometry prior to stroke. First, an initial linear 12 degree of freedom (dof) transform (Supplementary Figure 2: “L1”) is computed on the bias field corrected skull stripped T1w image (Supplementary Figure 2: image “4”) with FSL’s FLIRT(Jenkinson and Smith, 2001;Jenkinson et al., 2002) (Supplementary Figure 2: image “5”) and applied to the T1w image with skull (Supplementary Figure 2: image “6”). The linear transform serves as a starting point to the non-linear transform to avoid finding an incorrect minimum in the optimization.

To create the chimeric brain, the non-lesioned hemisphere’s tissue is “stitched” into the area of the lesion. First, a mirror image of the intact hemisphere is generated in MNI space by extracting the non-lesioned hemisphere (in this case the right hemisphere) from the linearly transformed T1w image with skull (Supplementary Figure 2: image “7”) and then flipped into the opposite hemisphere (Supplementary Figure 2: image “8”). The mirror image non-lesioned (right) hemispheres are “stitched” together (Supplementary Figure 2: image “9”), on which the first non-linear transform with FSL’s FNIRT (Supplementary Figure 2: “N1”) into MNI space is calculated (Supplementary Figure 2: image “10”). The N1 transform is applied to the lesion mask to estimate the lesion location in MNI space (Supplementary Figure 2: image “11”). Combining images 10 and 11, the tissue composition underneath the lesion is estimated (Supplementary Figure 2: image “12”). The estimated tissue composition is then back-transformed into native space using the inverse warp matrix (Supplementary Figure 2: image “13”) and replaces lesioned tissue from image 1 to generate the chimeric image (Supplementary Figure 2: image “14”). The chimera image does not contain signal changes due to the presence of a lesion, which reduces imperfections in the spatial normalization procedure.

Finally, the brain mask is applied on the chimera image (Supplementary Figure 2: image “15”) to compute the final linear transform (Supplementary Figure 2: “L2”) followed by the computation of the final non-linear transform (Supplementary Figure 2: “N2” and image “16”). To obtain the lesioned brain image 1 in MNI space, nonlinear transform N2 is applied using FSL’s applywarp (Supplementary Figure 2: image “17”). The T2w/T1w image is also transformed with “N2” using the FSL function applywarp.

It is important to note that using the non-linear transform “N1” will result in incorrect transforms of the lesioned hemisphere because the left and right hemisphere brains are different sizes (e.g. left frontal > right frontal, right parietal > left parietal), which are reflected in the MNI template brain and final calculated warp matrix “N2”.


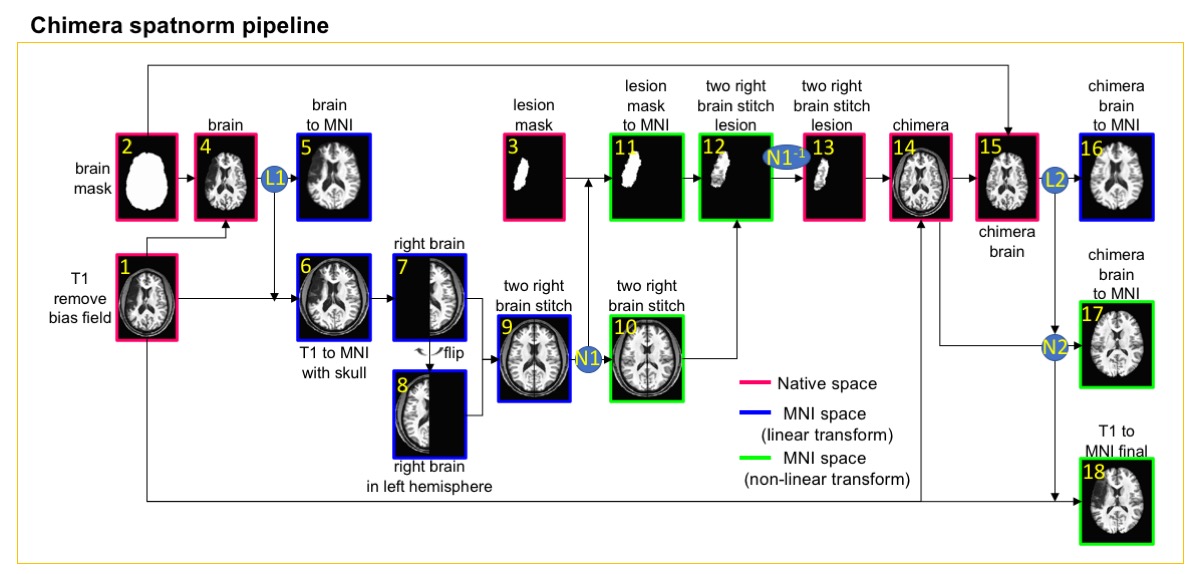


**Supplementary Figure 2:** Workflow of creating a chimeric brain to reduce imperfections in the non-linear transform into template space due to large cortical lesions.

*Contra-lesional hemisphere grey matter ribbon*

The eroded anterior contra-lesional hemisphere grey matter ribbon is extracted by choosing all non-zero eroded segmented GM voxels in an area bounded by x>0mm and y>15mm and all of z. The resulting area is shown in yellow in Supplementary Figure 3 and covers frontal lobe, anterior basal ganglia, and anterior temporal pole.


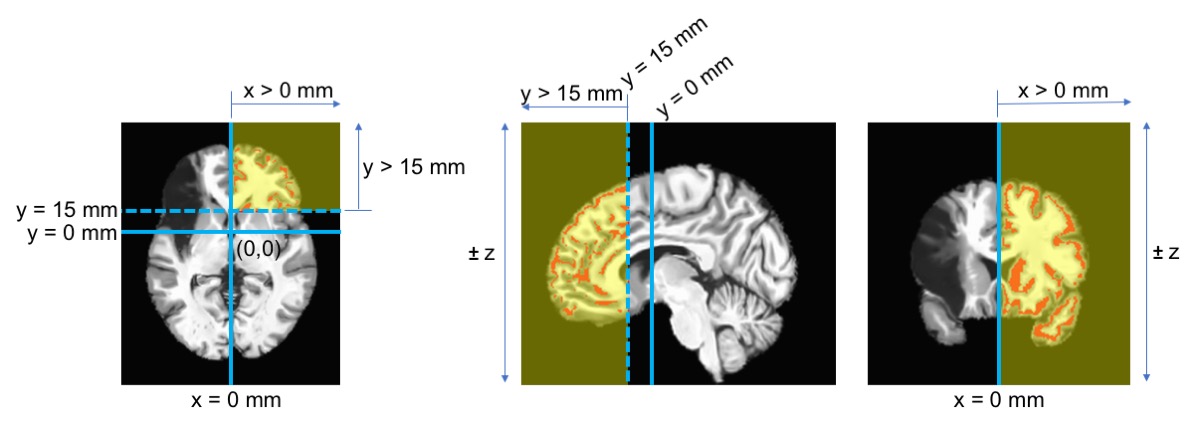


**Supplementary Figure** **3:** The yellow area shows the bounds of the anterior GM ribbon extracted for the TIGR GM threshold in axial, sagittal, and coronal views.

*LINDA lesion probability maps compared to TIGR maps and CBF relationship*

LINDA provides a lesion probability map in native space (Predict3_native.nii) that spatially identifies the probability of a lesion residing in a voxel. It is important to determine if TIGR and LINDA probability maps are similar or if TIGR provides additional information, especially because TIGR in this study was quantified within a lesion mask determined by LINDA. It is also important to determine if LINDA probability maps have a negative relationship with CBF (i.e., increase in lesion probability corresponds to a decrease in local blood flow), which is similar to the concept of TIGR-CBF relationship, where increase in tissue damage is related to a decrease in local CBF.

To perform the LINDA probability analysis to relate to CBF, we thresholded the Predict3_native.nii image into 4 areas: 0.1-0.39 (low probability), 0.4-0.79 (medium probability), 0.8-1.0 (high probability), and a perilesional area that represents a 10mm band around the probability map thresholded at 0.1. Similar to the TIGR analysis, the CBF in each probability zone is averaged and then entered into an ANOVA in JMP Pro15 to determine if lesion probability relates to regional CBF.

As seen visually in Supplementary Figure 4A, the TIGR maps are spatially more specific than the LINDA lesion probability maps. However, the ANOVA results do indicate that as the probability of lesion increases, the regional CBF significantly decreases (F(1,22)=14.4722, p=0.001). This finding is consistent with the TIGR-CBF relationship, but because TIGR score is spatially more confined to the high lesion probability areas, TIGR provides additional information. Furthermore, due to TIGR’s evaluation of highly spatially specific tissue damage and regional relationship to blood flow, the clinical utility of TIGR is likely to come in the form of targeted treatment planning, such as rTMS localization or tDCS high-definition electrode placement.


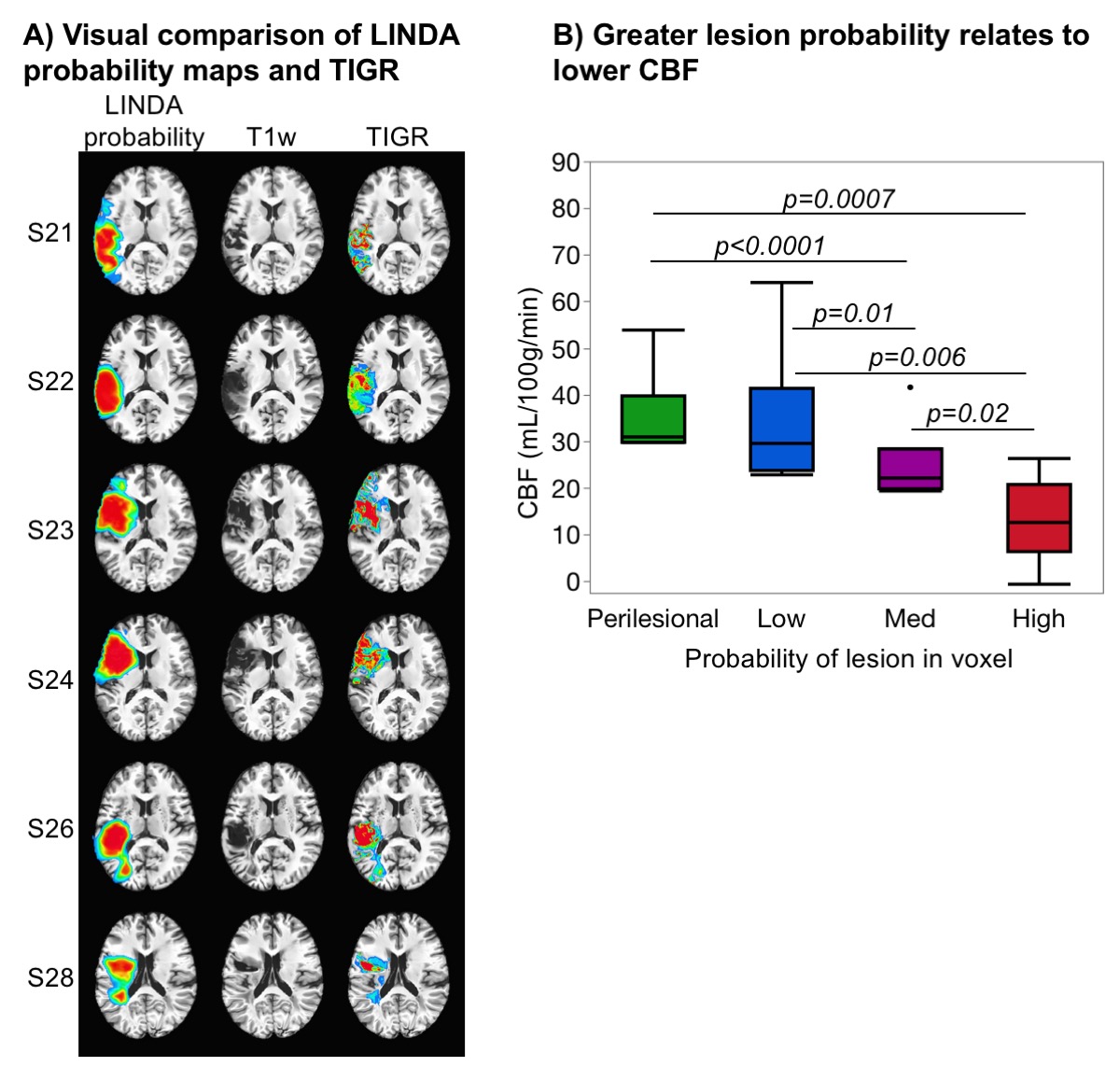


**Supplementary Figure** **4:**
(A) Visualization of LINDA probability maps with respect to source T1w images and corresponding TIGR maps. (B) A greater probability of lesion within a voxel corresponds to a reduced CBF.

*Individual participant’s task activation maps*

To validate TIGR’s ability to identify viable tissue, task fMRI was utilized to identify brain areas that were engaged during the task. Such brain areas include regions within the lesion. Shown here (Supplementary Figures 5-9) are the individual task activation maps with respect to TIGR maps for each participant.


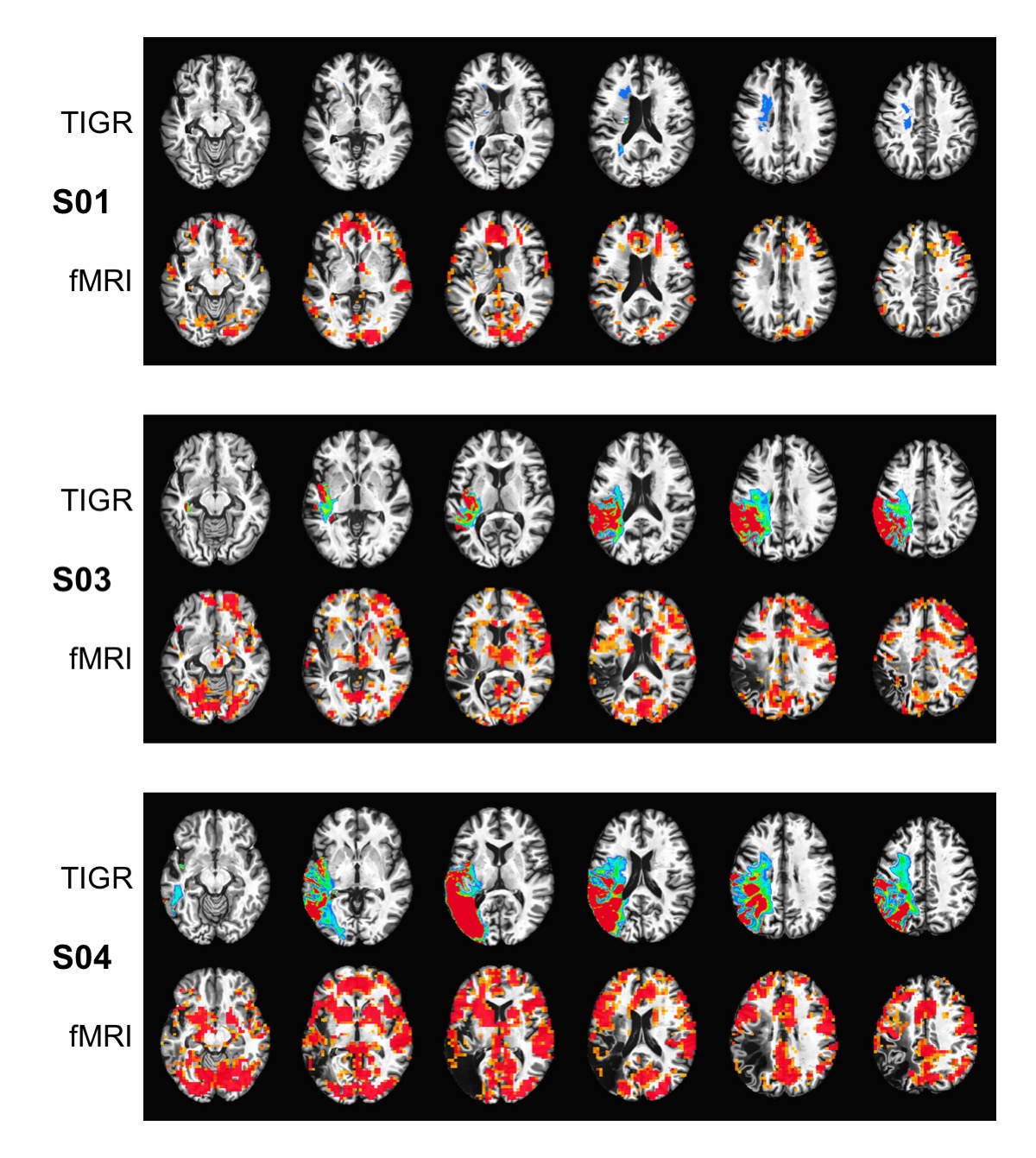


**Supplementary Figure 5:** TIGR maps and corresponding significant task activation for participants S01, S03, and S04.


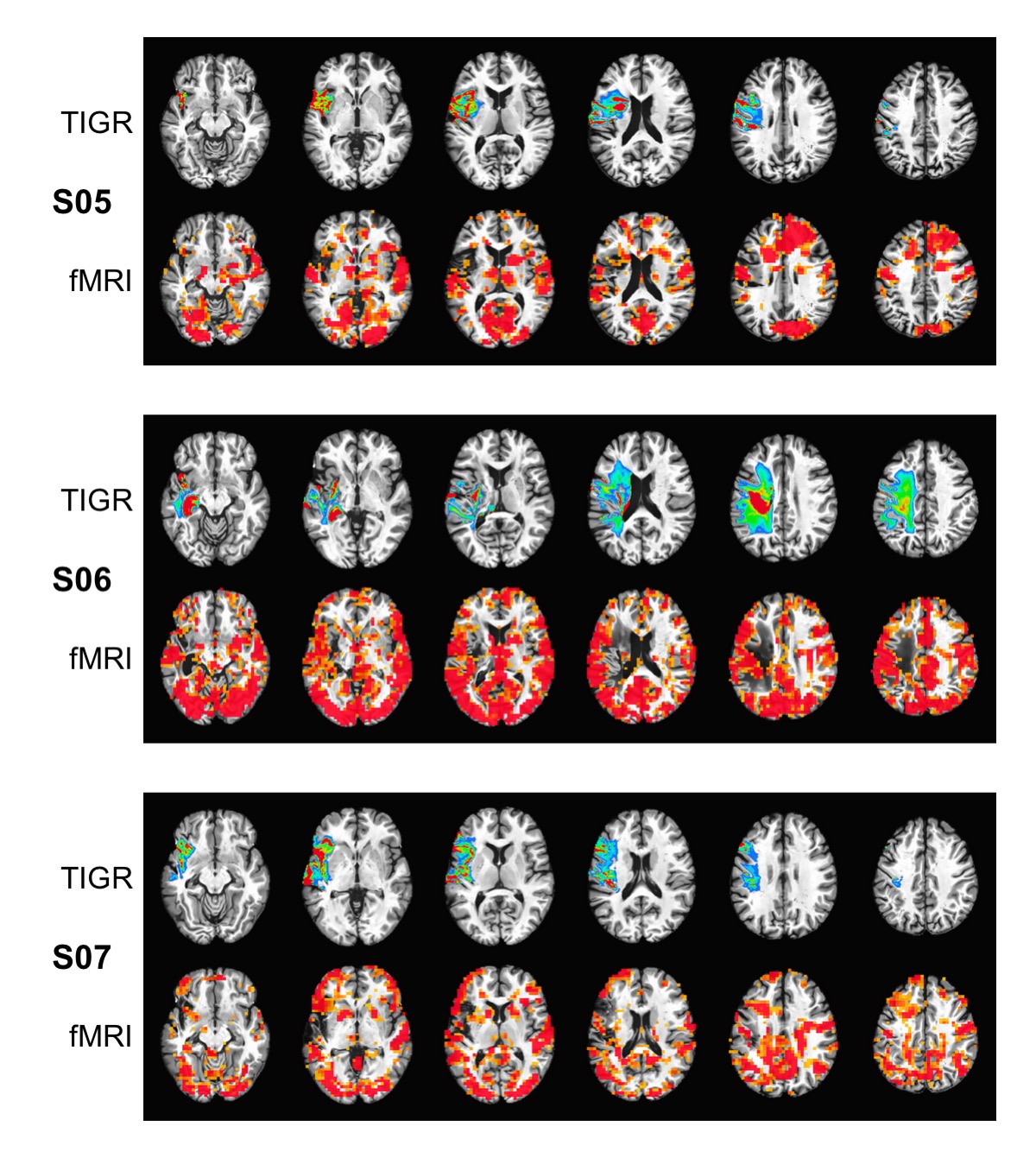


**Supplementary Figure 6:** TIGR maps and corresponding significant task activation for participants S05, S06, and S07.


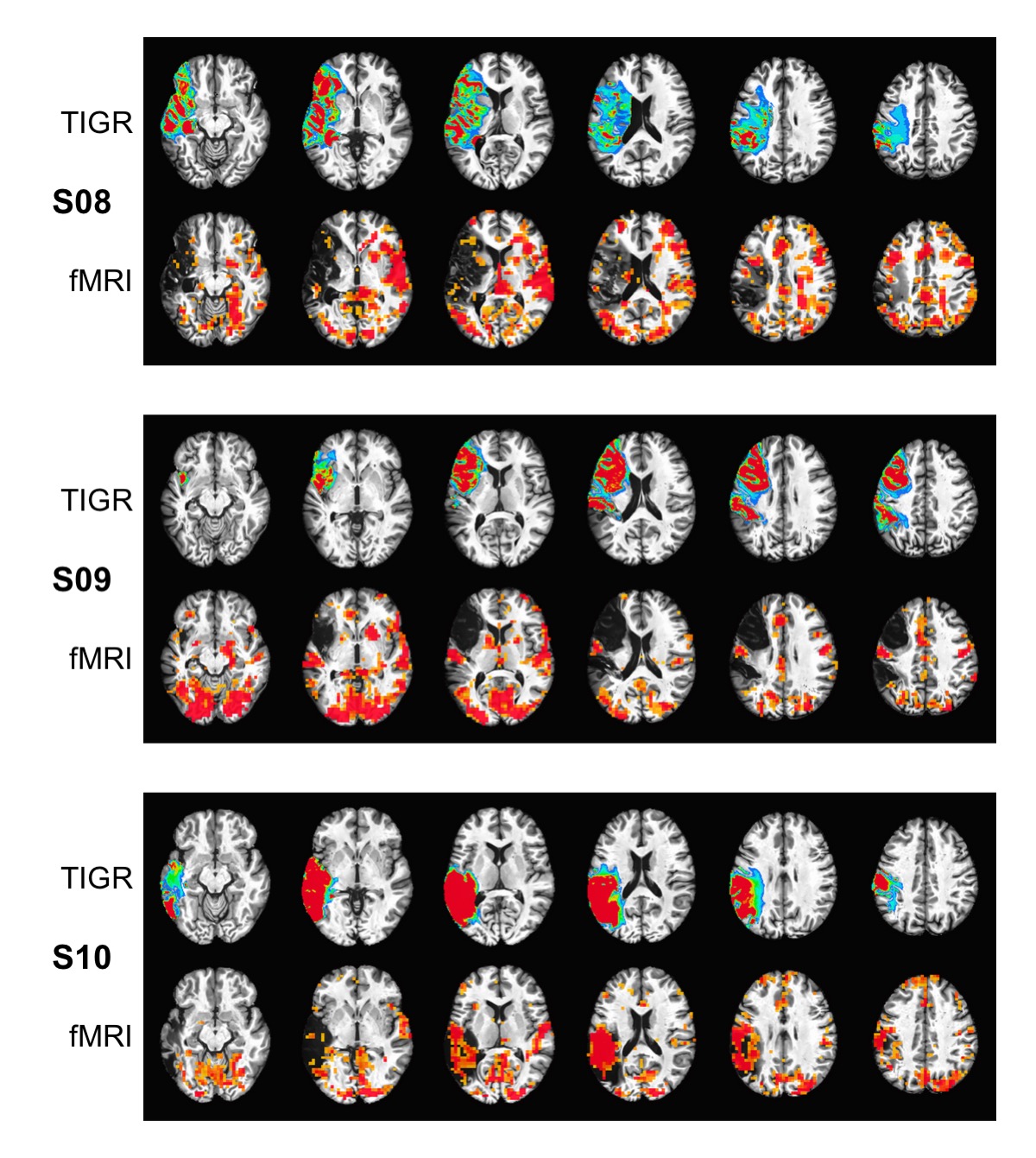


**Supplementary Figure 7:** TIGR maps and corresponding significant task activation for participants S08, S09, and S10.


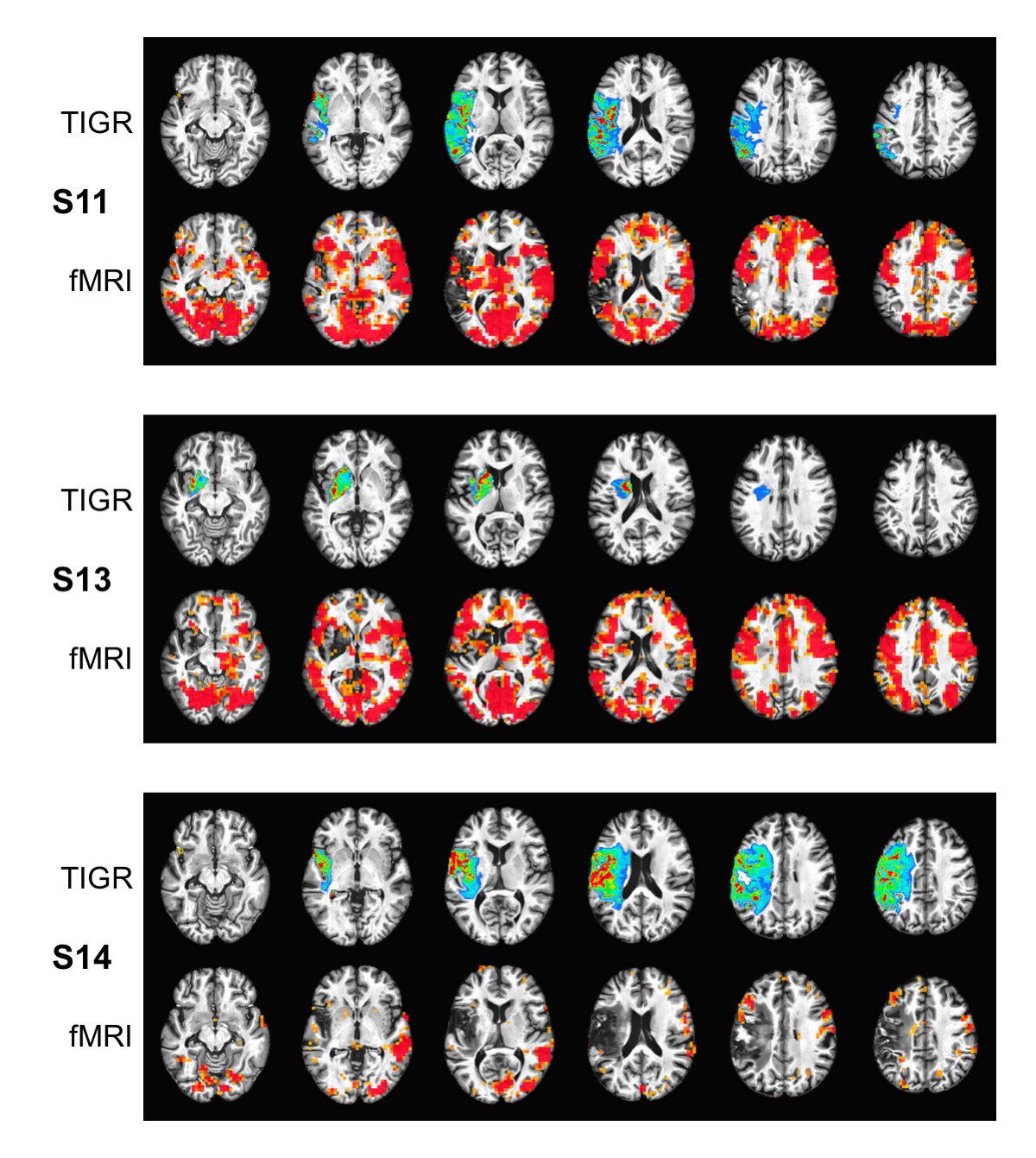


**Supplementary Figure 8:** TIGR maps and corresponding significant task activation for participants S11, S13, and S14.


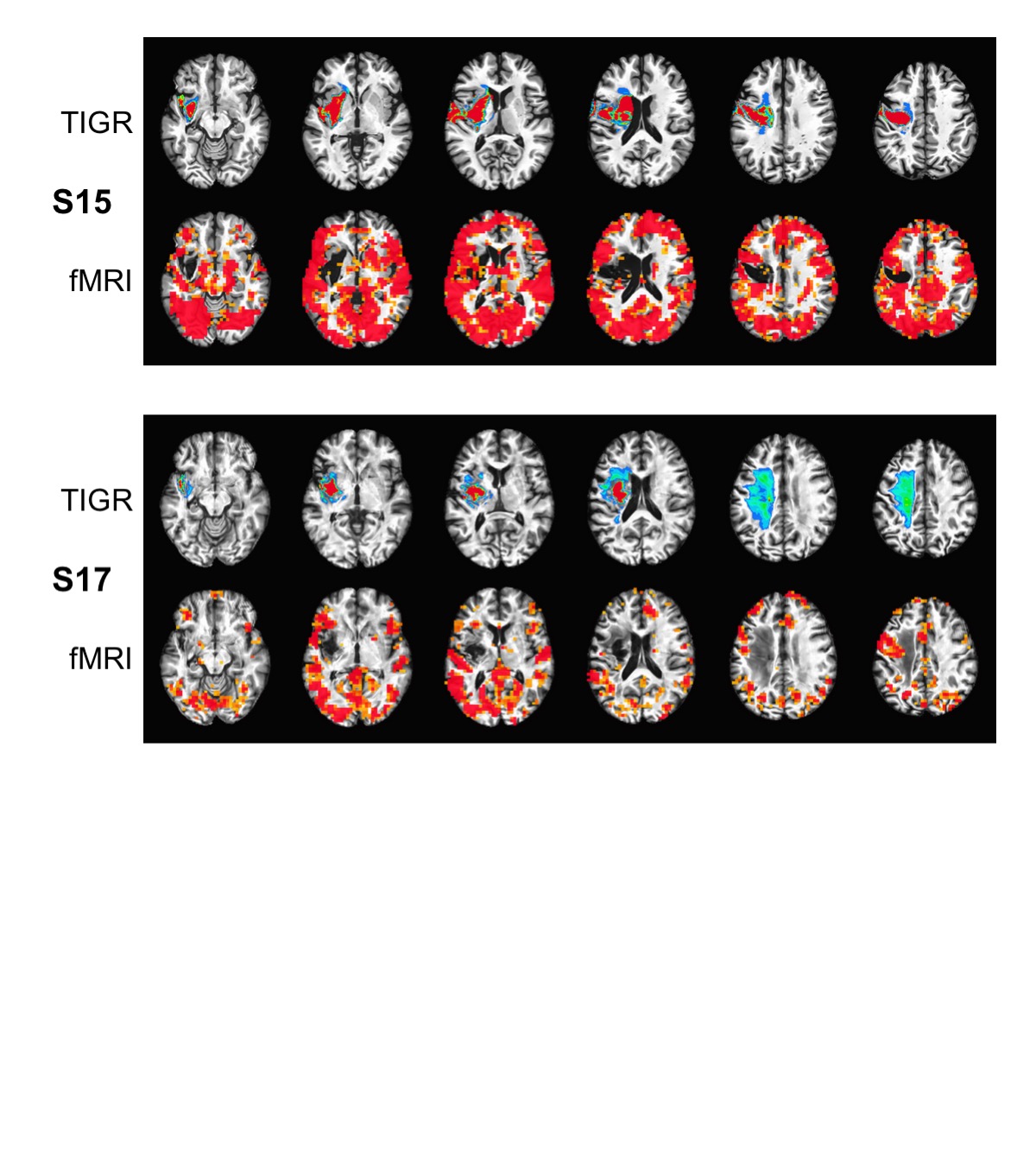


**Supplementary Figure 9:** TIGR maps and corresponding significant task activation for participants S15 and S17.

*Feasibility of quantifying TIGR in pontine and cerebellar lesions*

TIGR MRI was assessed on two participants (53 y.o Female and 53 y.o Male) with a pontine or cerebellar lesion obtained from a publicly available stroke dataset (Corbetta et al., 2015;Siegel et al., 2017). The T1w and T2w images were assessed for motion and then processed to quantify TIGR as described previously. Seen in Supplementary Figure *10* are the resulting chronic T2w, T1w, and TIGR maps from each participant. A gradient of tissue damage is observed, indicating that it is feasible to quantify TIGR in pontine and cerebellar lesions. More systematic work in this direction is required to determine if other steps are necessary to optimize the TIGR methodology for these regions.


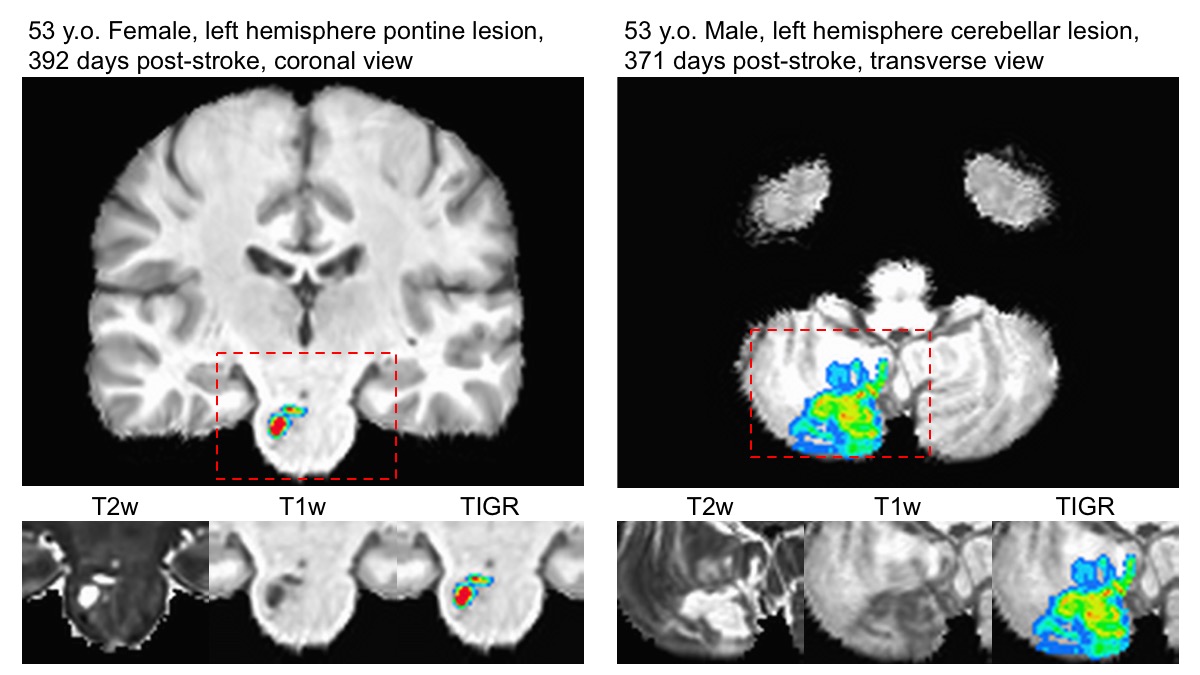


**Supplementary Figure 10:** Feasibility of TIGR maps in pontine lesion and cerebellar lesion.

**Supplementary Table 2: WAB auditory comprehension cluster locations and size**

| **Type** | **Cluster center of mass** | **Cluster size (1mm iso voxels)** | **Left hemisphere brain areas** |
| --- | --- | --- | --- |
| Overlap | X= -48.4, Y= -21.7, Z= +0.9  X= -33.5, Y= -37.6, Z= +24.5  X= -43.6, Y= -39.8, Z= +27.4  X= -51.3, Y= +10.8, Z= -20.7  X= -46.3, Y= -24.8, Z= +17.9  X= -29.2, Y= -51.4, Z= +53.9  X= -48.3, Y= +19.6, Z= -15.3 | 11360 voxels  1015 voxels  161 voxels  159 voxels  138 voxels  50 voxels  31 voxels | Heschl’s gyrus  Insula  Middle temporal gyrus  Superior temporal gyrus  Supramarginal gyrus  Arcuate fasciculus (WM)  Supramarginal gyrus  Superior temporal gyrus  Heschl’s gyrus  Superior parietal lobule  Temporal pole |
| Binary lesion | X= -44.4, Y= -27.3, Z= +6.2  X= -29.0, Y= -49.3, Z= +57.5 | 46406 voxels  4518 voxels | Angular gyrus  Arcuate fasciculus (WM)  Heschl’s gyrus  Insula  Middle temporal gyrus  Superior temporal gyrus  Supramarginal gyrus  Temporal pole  Temporal stem (WM)  Superior parietal lobule |
| TIGR | X= -45.0, Y= -25.7, Z= +6.5  X= -52.1, Y= +5.7, Z= +31.5  X= -27.5, Y= -50.3, Z= +56.3 | 41737 voxels  6323 voxels  3701 voxels | Angular gyrus  Arcuate fasciculus (WM)  Heschl’s gyrus  Insula  Middle temporal gyrus  Superior temporal gyrus  Supramarginal gyrus  Temporal pole  Pars opercularis  Precentral gyrus  Ventral premotor  Superior parietal lobule |
| T1w-only | X= -49.4, Y= -18.3, Z= -0.4  X= -32.9, Y= -35.8, Z= +24.5  X= -11.9, Y= -25.1, Z= +21.3  X= -50.7, Y= -25.1, Z= +21.3 | 20746 voxels  1146 voxels  640 voxels  555 voxels | Angular gyrus  Heschl’s gyrus  Insula  Middle temporal gyrus  Pars triangularis  Superior parietal lobule  Superior temporal gyrus  Supramarginal gyrus  Temporal pole  Arcuate fasciculus (WM)  Supplementary motor area  Postcentral gyrus |
| T2w-only | X= -44.1, Y= -21.7, Z= +4.8  X= -27.6, Y= -75.1, Z= +29.6 | 45558 voxels  560 voxels | Angular gyrus  Caudate  Heschl’s gyrus  Inferior temporal  Insula  Internal capsule (WM Superior) Middle temporal  Pars triangularis  Postcentral gyrus  Supramarginal gyrus  Superior parietal lobule  Superior temporal gyrus  Temporal gyrus  Temporal pole  Thalamus  Precuneus |

**Supplementary Table 3: WAB repetition cluster locations and size**

| **Type** | **Cluster center of mass** | **Cluster size (1mm iso voxels)** | **Left hemisphere brain areas** |
| --- | --- | --- | --- |
| Overlap | X= -48.8, Y= -23.3, Z= +2.1  X= -45.6, Y= -32.1, Z= +26.8  X= -55.0, Y= -14.7, Z= +23.2  X= -26.2, Y= -48.7, Z= +51.9  X= -36.3, Y= -54.4, Z= +58.4  X= -38.6, Y= -15.8, Z= -2.8  X= -37.6, Y= -48.7, Z= +59.2 | 6119 voxels  3893 voxels  550 voxels  356 voxels  58 voxels  56 voxels  31 voxels | Heschl’s gyrus  Superior temporal gyrus  Insula  Supramarginal gyrus  Postcentral gyrus  Superior parietal lobule  Superior parietal lobule  Insula  Superior parietal lobule |
| Binary lesion | X= -48.7, Y= -26.7, Z= +11.5  X= -29.8, X= -48.0, Z= +57.0 | 21515 voxels  2422 voxels | Heschl’s gyrus  Insula  Postcentral gyrus  Superior temporal gyrus  Supramarginal gyrus  Superior parietal lobule |
| TIGR | X= -49.8, Y= -26.2, Z= +13.6  X= -31.6, Y= -47.6, Z= +58.4 | 32908 voxels  3961 voxels | Heschl’s gyrus  Insula  Middle temporal gyrus  Postcentral gyrus  Precentral gyrus  Superior temporal gyrus  Supramarginal gyrus  Superior parietal lobule |
| T1w-only | X= -50.3, Y= -29.1, Z= +10.8  X= -25.8, Y= -45.6, Z= +52.8  X= -31.8, Y= +35.8, Z= -5.8  X= -27.4, Y= -80.3, Z= +13.2  X= -9.2, Y= -24.5, Z= +69.8  X= -57.6, Y= -2.0, Z= +25.9  X= -30.9, Y= +14.7, Z= +56.6  X= -22.3, Y= -8.3, Z= +41.7  X= -31.3, Y= +51.6, Z= +17.2  X= -25.5, Y= -17.6, Z= +69.5  X= -39.9, Y= +8.9, Z= +51.2 | 48369 voxels  10175 voxels  5814 voxels  1890 voxels  1402 voxels  985 voxels  845 voxels  792 voxels  717 voxels  701 voxels  525 voxels | Heschl’s gyrus  Inferior temporal gyrus  Insula  Middle temporal gyrus  Postcentral gyrus  Superior temporal gyrus  Supramarginal gyrus  Precentral gyrus  Precuneus  Superior parietal lobule  Anterior thalamic radiation (WM)  Pars orbitalis  Middle occipital gyrus  Precentral gyrus  Precentral gyrus  Dorsal premotor  Arcuate fasciculus (WM)  Middle frontal gyrus  Precentral gyrus  Dorsal premotor |
| T2w-only | X= -45.1, Y= -22.7, Z= +11.5  X= -27.6, Y= -50.7, Z= +50.6  X=-44.3, Y= +14.2, Z= -7.8  X= -35.3, Y= -32.4, Z= +50.7 | 22839 voxels  2062 voxels  651 voxels  585 voxels | Heschl’s gyrus  Insula  Internal capsule (WM)  Middle temporal gyrus  Postcentral gyrus  Superior temporal gyrus  Supramarginal gyrus  Superior parietal lobule  Pars triangularis  Superior temporal lobe  Postcentral gyrus |

**Supplementary Table 4: WAB spontaneous speech cluster locations and size**

| **Type** | **Cluster center of mass** | **Cluster size (1mm iso voxels)** | **Left hemisphere brain areas** |
| --- | --- | --- | --- |
| Overlap | X= -47.1, Y= -39.7, Z= +34.6  X= -52.2, Y= -23.9, Z=21.6  X= -39.4, Y= -5.4, Z= -3.9  X= -31.6, Y= -47.1, Z= +59.6  X= -47.6, Y= +6.5, Z= -8.6  X= -23.0, Y= +1.2, Z= +24.5  X= -26.5, Y= -13.3, Z= +25.0  X= -48.5, Y= -45.8, Z= +50.7  X= -15.3, Y= -15.4, Z= +18.5  X= -35.5, Y= -54.1, Z= +57.8  X= -34.3, Y= -11.0, Z= -8.2 | 4480 voxels  1413 voxels  782 voxels  615 voxels  467 voxels  345 voxels  180 voxels  99 voxels  42 voxels  39 voxels  34 voxels | Supramarginal gyrus  Heschl’s gyrus  Insula  Superior parietal lobule  Superior temporal gyrus  Arcuate fasciculus (WM)  Arcuate fasciculus (WM)  Supramarginal gyrus  Thalamus  Superior parietal lobule  Temporal stem (WM) |
| Binary lesion | X= -42.2, Y= -28.5, Z= +28.8  X= -45.6, Y= -46.8, Z= -0.9 | 78693 voxels  1268 voxels | Arcuate fasciculus (WM)  Caudate  External capsule (WM)  Globus pallidus  Heschl’s gyrus  Inferior frontal gyrus  Insula  Internal capsule (WM)  Middle temporal gyrus  Postcentral gyrus  Precentral gyrus  Putamen  Superior parietal lobule  Superior temporal gyrus  Supramarginal gyrus  Temporal pole  Thalamus  Temporal stem (WM) |
| TIGR | X= -48.7, Y= -36.6, Z= +32.5  X= -42.8, Y= -0.2, Z= -7.0  X= -31.4, Y= -49.6, Z= +62.5  X= -24.5, Y= -6.4, Z= +25.1 | 9014 voxels  2424 voxels  2046 voxels  902 voxels | Heschl’s gyrus  Supramarginal gyrus  Insula  Superior Temporal gyrus  Temporal stem (WM)  Uncinate fasciculus (WM)  Superior parietal lobule  Thalamus  Arcuate fasciculus (WM) |
| T1w-only | X= -44.0, Y= -23.4, Z= +20.8  X= -59.8, Y=-29.3, Z= -11.9  X= -17.3, Y= +4.1, Z= +19.9  X= -23.5, Y= -61.5, Z= +45.7  X= -33.7, Y= -28.7, Z= -8.9  X= -25.3, Y= -12.2, Z= +62.7  X= -47.7, Y= +8.4, Z= +33.2 | 29996 voxels  8088 voxels  2055 voxels  1278 voxels  1191 voxels  879 voxels  586 voxels | Arcuate fasciculus (WM)  Caudate  External capsule (WM)  Heschl’s gyrus  Insula  Middle temporal gyrus  Pars orbitalis  Pars triangularis  Postcentral gyrus  Superior parietal lobule  Superior temporal gyrus  Supramarginal gyrus  Temporal pole  Temporal stem (WM)  Thalamus  Inferior temporal  Middle temporal  Arcuate fasciculus (WM)  Caudate  Superior parietal lobule  Hippocampus  Dorsal premotor  Ventral premotor |
| T2w-only | X= -41.0, Y= -15.6, Z= +12.7  X= -47.5, Y=-70.7, Z= +33.8  X= -22.7, Y= -29.6, Z= +65.9  X= -31.0, Y= -76.7, Z= +39.2 | 126115 voxels  1563 voxels  1312 voxels  1211 voxels | Arcuate fasciculus (WM)  Caudate  Dorsal premotor  Dorsolateral prefrontal cortex  External capsule (WM)  Globus pallidus  Heschl’s gyrus  Hippocampus  Inferior temporal  Insula  Internal capsule (WM)  Middle temporal  Pars orbitalis  Pars triangularis  Precentral gyrus  Precuneus  Postcentral gyrus  Putamen  Superior parietal lobule  Superior temporal  Supramarginal gyrus  Temporal pole  Temporal stem (WM)  Thalamus  Ventral premotor  Angular gyrus  Postcentral gyrus  Precuneus |

**Supplementary Materials References**

Corbetta, M., Ramsey, L., Callejas, A., Baldassarre, A., Hacker, C.D., Siegel, J.S., Astafiev, S.V., Rengachary, J., Zinn, K., Lang, C.E., Connor, L.T., Fucetola, R., Strube, M., Carter, A.R., and Shulman, G.L. (2015). Common behavioral clusters and subcortical anatomy in stroke. *Neuron* 85**,** 927-941.

Jenkinson, M., Bannister, P., Brady, M., and Smith, S. (2002). Improved optimization for the robust and accurate linear registration and motion correction of brain images. *Neuroimage* 17**,** 825-841.

Jenkinson, M., and Smith, S. (2001). A global optimisation method for robust affine registration of brain images. *Med Image Anal* 5**,** 143-156.

Lutkenhoff, E.S., Rosenberg, M., Chiang, J., Zhang, K., Pickard, J.D., Owen, A.M., and Monti, M.M. (2014). Optimized brain extraction for pathological brains (optiBET). *PLoS One* 9**,** e115551.

Pustina, D., Coslett, H.B., Turkeltaub, P.E., Tustison, N., Schwartz, M.F., and Avants, B. (2016). Automated segmentation of chronic stroke lesions using LINDA: Lesion identification with neighborhood data analysis. *Hum Brain Mapp* 37**,** 1405-1421.

Siegel, J.S., Shulman, G.L., and Corbetta, M. (2017). Measuring functional connectivity in stroke: Approaches and considerations. *J Cereb Blood Flow Metab* 37**,** 2665-2678.

Smith, S.M., Jenkinson, M., Woolrich, M.W., Beckmann, C.F., Behrens, T.E., Johansen-Berg, H., Bannister, P.R., De Luca, M., Drobnjak, I., Flitney, D.E., Niazy, R.K., Saunders, J., Vickers, J., Zhang, Y., De Stefano, N., Brady, J.M., and Matthews, P.M. (2004). Advances in functional and structural MR image analysis and implementation as FSL. *Neuroimage* 23 Suppl 1**,** S208-219.

Yourganov, G., Fridriksson, J., Stark, B., and Rorden, C. (2018). Removal of artifacts from resting-state fMRI data in stroke. *Neuroimage Clin* 17**,** 297-305.

Yushkevich, P.A., Piven, J., Hazlett, H.C., Smith, R.G., Ho, S., Gee, J.C., and Gerig, G. (2006). User-guided 3D active contour segmentation of anatomical structures: significantly improved efficiency and reliability. *Neuroimage* 31**,** 1116-1128.

Zhang, Y., Brady, M., and Smith, S. (2001). Segmentation of brain MR images through a hidden Markov random field model and the expectation-maximization algorithm. *IEEE Trans Med Imaging* 20**,** 45-57.
